# *Pseudomonas halotolerans* sp. nov., a halotolerant biocontrol agent with plant-growth properties

**DOI:** 10.3389/fpls.2025.1605131

**Published:** 2025-05-21

**Authors:** Patricia Sánchez, Inés Castillo, Fernando Martínez-Checa, Inmaculada Sampedro, Inmaculada Llamas

**Affiliations:** ^1^ Department of Microbiology, Faculty of Pharmacy, University of Granada, Granada, Spain; ^2^ Biomedical Research Centre (CIBM), Institute of Biotechnology, University of Granada, Granada, Spain

**Keywords:** *Pseudomonas*, plant-growth promoting bacteria, quorum quenching, phytopathogen, halotolerant bacteria

## Abstract

A polyphasic taxonomic approach was conducted to characterize the bacterial strain B22^T^ isolated from the rhizospheric soil of the halophyte *Salicornia hispanica*. This strain is aerobic, Gram-negative, rod-shaped, catalase and oxidase positive, motile, reduces nitrates and chemoheterotrophic. It is halotolerant, exhibiting optimal growth at 28°C and pH 7.0 in the presence of 0.5-2.5% (w/v) of NaCl. The B22^T^ genome size is 5.7 Mbp, with a G+C content of 60.5 mol%. This strain has the capacity to promote tomato growth by producing siderophores, indole-3-acetic acid and enzymes such as phytase and acid phosphatase. Additionally, strain B22^T^ produces a quorum quenching (QQ) enzyme capable of degrading synthetic *N*-acylhomoserine lactones (AHLs) as well as those produced by phytopathogens. The interference of plant pathogen communication reduced virulence in tomato fruits and plants. Phylogenetic analysis revealed that the closest relatives of strain B22^T^ was *Pseudomonas tehranensis* SWRI 196^T^. The average nucleotide identity values between strain B22^T^ and *P. tehranensis* SWRI 196^T^ was 95.1% while digital DNA-DNA hybridization values was 64.5% The main cellular fatty acids of strain B22^T^ were C_16:0_, summed feature 3 (C_16:1_ *ω*7*c*/C_16:1_ *ω*6*c*) and summed feature 8 (C_18:1_ *ω*7*c*/C_18:1_ *ω*6*c*). The major polar lipids identified were diphosphatidylglycerol and phosphatidylethanolamine, while the predominant respiratory quinone was ubiquinone (Q-9). Based on genomic, phylogenetic and chemotaxonomic data, strain B22^T^ (=CECT 31209; =LMG33902) represents a novel species within the genus *Pseudomonas.* The name *Pseudomonas halotolerans* sp. nov. is proposed. Additionally, this study highlights the potential of *P. halotolerans* as a sustainable biocontrol agent due to its plant growth-promoting activity in tomato plants and its ability to reduce phytopathogen virulence factors, mitigating damage to fruits and plants.

## 1 Introduction

Global agriculture faces a complex array of challenges as it strives to feed a growing population, projected to reach nearly 10 billion by 2050 (FAO, 2018). Intensive farming practices, combined with the widespread use of chemical fertilizers and pesticides, have led to significant environmental degradation, including pollution, loss of beneficial soil microbes, and reduced biodiversity (Hartmann et al., 2015; Kopittke et al., 2019; Tripathi et al., 2020). One of the most serious threats to crop productivity and food security worldwide is the spread of pest and plant diseases. The misuse and overuse of agrochemicals have significantly contributed to the development of resistant phytopathogens, further complicating efforts to manage plant diseases (FAO, 2023). Addressing these challenges is a priority in European agricultural policies, which emphasize the need for sustainable alternatives to traditional, chemical-based practices. These approaches aim to protect ecosystems while supporting food security (Schneider et al., 2023; Finger and Möhring, 2024; FAO, 2023).

Beneficial plant associated microorganisms are well-documented as an eco-friendly strategy to reduce the use of agrochemical compounds due to their capability of promoting plant growth (plant growth-promoting bacteria, PGPB) and/or protecting against phytopathogens (biological control agents, BCAs) (Berg et al., 2017; Singh, 2018; Jiao et al., 2021).

Regarding to *Pseudomonas* genus, several plant-associated species have been recognized for their beneficial interaction with plants and their role in the biocontrol of phytopathogens (Weller, 2007; Navarro-Monsterrat and Taylor, 2023). Such is the case of *P. chlororaphis* (Arrebola et al., 2019; Raio and Puopolo, 2021), *P. fluorescens* (Al-Karablieh et al., 2022), *P. protegens* (Bakaeva et al., 2022; Ramette et al., 2011), *P. putida* (Weimer et al., 2020; Costa-Gutiérrez et al., 2022), and *P. segetis* (Rodríguez et al., 2020). These species exhibit a range of beneficial traits, including nutrient solubilization, phytohormone production, nitrogen fixation and induction of systemic resistance (ISR). Their biocontrol capabilities are attributed to diverse mechanisms, such as the production of bioactive metabolites like phenazines, pyoverdines, cyclic lipopeptides and volatile compounds with antimicrobial activities (Raio and Puopolo, 2021; Al-Karablieh et al., 2022; Balthazar et al., 2022; Ajijah et al., 2023; Navarro-Monsterrat and Taylor, 2023). Additionally, the remarkable adaptability of *Pseudomonas* strains to diverse environmental conditions enables effective root colonization and competitive exclusion of pathogens. Together, these traits contribute to improve plant health and resilience (Garrido-Sanz et al., 2023).

Among the mechanisms for biocontrol, the disruption of quorum sensing (QS) systems in plant pathogens has emerged as a promising, eco-friendly alternative for managing plant diseases, reducing reliance on agrochemicals (Defoirdt, 2018; Verma et al., 2021; Sharma et al., 2022; Zhu et al., 2023). QS is a cell-density-dependent communication system mediated by the diffusion of signaling molecules, such as N-acylhomoserine lactones (AHLs) in Gram-negative bacteria, and coordinates behaviors critical to pathogenicity (Baltenneck et al., 2021; Von Bodman et al., 2003). These QS-regulated traits include the production of hydrolytic enzymes, biofilm formation, toxin production, and host invasion, as observed in phytopathogens such as *Dickeya solani* (Crépin et al., 2012; Potrykus et al., 2018), *Erwinia amylovora* (Venturi et al., 2004; Piqué et al., 2015), *Pantoea agglomerans* (Cruz et al., 2024), *Pectobacterium atrosepticum* (Smadja et al., 2004), *P. carotovorum* (Moleleki et al., 2017; Pollumaa et al., 2012) and *Pseudomonas syringae* (Quinones et al., 2005; Cheng et al., 2016).

One extensively studied mechanisms to interfere QS is quorum quenching (QQ), which involves the enzymatic degradation or modification of AHLs via lactonases, acylases, or oxidoreductases (Grandclément et al., 2016). Studies have demonstrated that heterologous expression of QQ enzymes in bacterial phytopathogens or co-cultivation with AHL-degrading bacteria reduces virulence, often resulting in decreased infection severity (Zhou et al., 2022; Zhu et al., 2023). In the case of *Pseudomonas* species, the strains *P. segetis* P6, *P. nitroreducens* W-7 and *P. multirresinivorans* QL-9a have reported to attenuate bacterial phytopathogen virulence through QQ mechanisms (Rodríguez et al., 2020; Liu et al., 2023).

This study aimed to achieve several objectives. The primary aim was to taxonomically identify the strain B22, which was isolated from the rhizospheric soil of a halophyte plant in the El Saladar de El Margen, Granada, southern Spain. Furthermore, this study analyzed its PGP and QQ activities and investigated its potential application as biocontrol agent. These properties underscore its suitability for integration into sustainable agricultural practices.

## 2 Material and methods

### 2.1 Bacterial strains, media, compounds and culture conditions

Strain B22^T^ was isolated from the rhizospheric soil of *Salicornia hipanica* in El Saladar del Margen, Cúllar, Granada, Spain (37° 38’ 50.6’’N, 2° 37’ 22.2’’ W). *Pseudomonas tehranensis* SWRI 196^T^ (LMG 32044), *P. alvandae* SWRI 17^T^ (LMG 32056) and *P. canavaninivorans* HB 002^T^ (LMG 32336) were used for taxonomic identification. The AHL-producing plant pathogenic strains used in this work were *Dickeya solani* LMG 25993^T^, *Pectobacterium atrosepticum* CECT 314^T^, *P. carotovorum* subsp. *carotovorum* CECT 225^T^, *Pseudomonas syringae* pv. tomato DC3000 and *Pantoea agglomerans* CFBP 11141. All of them were routinely grown in Luria Bertani (LB) media at 28°C.

The AHL biosensor strains *Chromobacterium subtsugae* CV026 (Harrison and Soby, 2020) and *C. violaceum* VIR07 (Morohoshi et al., 2008) were grown in LB medium while *Agrobacterium tumefaciens* NTL4 (pZLR4) (Shaw et al., 1997) was cultured in LB medium supplemented with 2.5 mmol L^-1^ CaCl_2_ x 2H_2_0 and 2.5 mmol L^-1^ MgSO_4_ x 7H_2_0 (LB/MC) or *Agrobacterium* broth (AB) medium (Chilton et al., 1974). The antibiotics kanamycin (Km) and gentamicin (Gm) were used in final concentrations of 50 μg mL^−1^ for the growth of the biosensor strains. All strains were grown at 28°C and at 120 rpm in an orbital shaker.

### 2.2 Phylogenetic 16S rRNA gene analysis

Genomic DNA of strain B22^T^ was extracted using the X-DNA Purification Kit (Xtrem Biotech S.L., Granada, Spain). The 16S rRNA gene was then amplified using universal bacterial primers 16F27 and 16R1488. The resulting PCR product was purified and cloned into the pGEM^®^-T vector (Promega).

Direct sequencing of the PCR-amplified DNA was performed, and the DNA sequence was compared with reference 16S rRNA gene sequences from GenBank and EMBL databases via the NCBI Genome Database using EzBioCloud server (Yoon et al., 2017).

Phylogenetic analysis was conducted using the Molecular Evolutionary Genetics Analysis (MEGA) software version X (Kumar et al., 2018), incorporating multiple sequence alignments with CLUSTAL OMEGA (Sievers et al., 2011). Evolutionary distances and clustering were determined using the neighbor-joining and maximum-likelihood methods. Cluster stability was assessed through bootstrap analysis with 1,000 replications.

### 2.3 Multilocus sequence analysis

To construct a more robust phylogenetic tree, a multigene approach was used. MLSA was conducted by concatenating the sequences of four housekeeping genes (16S *rRNA, gyrB, rpoD*, and *rpoB*). Housekeeping gene sequences from other *Pseudomonas* species were retrieved from the genomes deposited in NCBI GenBank database. Multiple sequence alignment of the concatenated gene sequences was performed using the MUSCLE algorithm embedded in MEGA X, followed by manual verification to identify and correct alignment errors.

A phylogenetic tree was generated using the neighbor-joining (NJ) algorithm, with 1000 bootstrap replicates to assess the robustness of the branching.

### 2.4 Genome sequencing and assembly

Genomic DNA of strain B22^T^, which was extracted as described above, was sequenced by the Illumina MiSeq methodology at the STAB VIDA facility (Caparica, Portugal) with 2 x 150-bp paired-end reads. The reads were trimmed using software tools implemented in the BBMap project (https://sourceforge.net/project/bbmap/) (Bushnell, 2016) to remove the adapters and low-quality bases and *de novo* assembled using SPADES v3.11.1 (Bankevich et al., 2012). CheckM v1.0.18 (Parks et al., 2015) and Quast v5.0.2 (Gurevich et al., 2013) were used for assembly quality checked. The genome of strain B22^T^ was annotated using RASTtk v1.073 (Aziz et al., 2008; Overbeek et al., 2014; Brettin et al., 2015) and deposited in GenBank/EMBL/DDBJ under the accession number JBCNTJ010000000.

### 2.5 *In silico* ANI and DDH

Average nucleotide identity (ANI) was determined by using OrthoANI software (Lee et al., 2016). For digital DNA–DNA hybridization (dDDH), values were calculated using the BLAST+ algorithm via the DSMZ Genome-to-Genome Distance Calculator (GGDC 3.0) web service at http://ggdc.dsmz.de/ (Meier-Kolthoff et al., 2013, 2022). The results presented in this study are based on the recommended Formula 2 (identities/HSP length), which is independent of genome length, ensuring robustness even when using incomplete draft genomes. The results of the strain described in this study were additionally validated using the Type (Strain) Genome Server [TYGS (Meier-Kolthoff et al., 2022)] in January 2025.

### 2.6 Analysis of the core orthologous genes

A core genome analysis of strain B22^T^ and 14 closest related bacteria based on their 16S rRNA percentage of similarity for which their genome was available, downloaded from the NCBI RefSeq database, was also performed using Bacterial Pan Genome Analysis (BPGA) software (Chaudhari et al., 2016) with the default parameters. After obtaining the core of these 15 bacterial genomes, all protein orthologs belonging to the core genome were concatenated and aligned by MAFFT (Katoh and Standley, 2013). A phylogenomic tree of the core genes of the species was then constructed using MEGA X software according to the maximum-likelihood method, incorporating 1,000 bootstrap replicates to evaluate the robustness of the branching.

### 2.7 Phenotypic and chemotaxonomic characterization

The colonies were observed on LB agar after 48 h of incubation at 28°C. The oxidase and catalase were determined. Salinity tolerance and optimal growth conditions of strain B22^T^ were determined at 28°C on LB agar plates supplemented with 0; 0.5; 2.5; 5; 7.5; 12 and 25% (w/v) of NaCl adjusting the pH 7. The pH growth range and optimum pH were analyzed on LB agar plates, testing from 4 to 10 pH unit intervals. The temperature range for growth and the optimum temperature were determined on LB plates at 4; 10; 15; 20; 25; 30; 37 and 40°C. In both cases 1% NaCl w/v was selected.

Phenotypic characteristics of strain B22^T^ related with the *in vitro* plant-growth-promoting (PGP) activities and rhizosphere competence were evaluated: amylase (Barrow and Feltham, 1993), caseinase (Barrow and Feltham, 1993), cellulase (Tasse et al., 2010), acid phosphatase (Baird-Parker, 1963), alkaline phosphatase (Pikovskaya, 1948), phytase (Hosseinkhani and Hosseinkhani, 2009), gelatinase (Tindall et al., 2007), lecithinase (Larpent and Larpent-Gourgand, 1957), quitinase, hydrolysis of Tween 20 and Tween 80 (Sierra, 1957), production of indole-3-acetic (IAA) (Naik et al., 2008) and siderophores (Alexander and Zuberer, 1991).

Other biochemical tests were performed using inoculum of B22^T^ grown in optimal conditions in API 20NE, API 50CH, API ZYM and BIOLOG GEN III according to the manufacturer’s instructions. In addition, DNAase (Jeffris et al., 1957) and hemolysin (Columbia blood agar plates, Difco) were detected.

The cellular fatty acids were analyzed at the Spanish Type Culture Collection (CECT) in Valencia, Spain, following the instruction of the Microbial Identification System Operating Manual (MIDI, 2008). For this, the cell mass of the B22^T^ strain was obtained after growing for 24 h in LB at 28°C and 1% NaCl w/v.

The analysis of polar lipids and respiratory quinones in strain B22^T^ was performed by the Identification Service at DSMZ in Braunschweig, Germany.

### 2.8 *In vivo* Plant-growth-promoting and colonization assays

To test the PGP activity of B22^T^, an *in vivo* assay was performed by inoculating tomato plants (*Solanum lycopersicum* L. var. Roma) with this bacterium. After the sterilization of the seeds, they were planted in seedbeds (20 x 7 cm) using sterile vermiculite as a substrate. Sterile water was added until the field capacity was reached, and it was covered with plastic film to maintain humidity. The seedlings were maintained in a greenhouse throughout the duration of the experiment, with a relative humidity (RH) of 60%, temperatures of 25°C during the day and 20°C at night, and a long-day photoperiod (light:dark, 16:8 hours). During a month, the plant seedlings were irrigated weekly with 250 μL of a bacterial suspension (10^8^ CFU mL^-1^), while the control group was irrigated with sterile distilled water. The experiment was carried out with 12 replicates. Once the treatment had concluded, the plants were harvested and the length, dry weight and thickness of the stem were determined (Sánchez et al., 2023).

Another assay to investigate the colonization properties of B22^T^ was also performed using the model plant *Arabidopsis thaliana* (Lee et al., 2020; Furci et al., 2021). Five sterile seeds were placed 2 cm apart in a square Petri dish (10 x 10 cm) containing Murashige & Skoog medium at 0.8% (w/v) agar and a final sucrose concentration of 2.5 g L^-1^. *In vitro* cultures were maintained under controlled conditions in a phytotron (Hederahelix Ing.) with a long day photoperiod (light:dark, 16:8 h), at 25°C day and 20°C night, relative humidity 60-80% and a luminosity of 250 microsiemens/cm^2^/sg. After one week of growth, the seedlings were inoculated with 1 μL of the bacterial suspension (10^8^ CFU mL^−1^) at 1 cm from the base of the plant stem. The same volume of sterile distilled water was inoculated in the control and three replicates were made per treatment. The plates were maintained under controlled conditions as mentioned above for another 7 days. After this period, root colonization was observed by differential interference contrast microscopy between slide and coverslip, using 70% (v/v) glycerol as mounting medium in a LEICA DM5500B microscope.

### 2.9 Quorum-quenching activity: range of AHLs, identification and localization of the enzyme

The QQ activity of the B22^T^ strain against synthetic AHLs was assessed through a well-diffusion agar-plate assay (Torres et al., 2013). In brief, each AHL was added to an overnight culture in LB medium of the B22^T^ strain, at a final concentration of 10μM, followed by a 24-hour incubation at 28°C. A sterile LB medium supplemented with AHLs served as negative control. The remaining AHLs were identified in the supernatant of each sample, which was then placed in wells on LB agar plates covered with *C. subtsugae* CV026 or *C. violaceum* VIR07, or on AB agar plates added with 80 μg mL^−1^ of 5-bromo-4-chloro-3-indolyl-ß-D-galactopyranoside (Xgal) and overlaid with *A. tumefaciens* NTL4 (pZLR4). The plates were then incubated at 28°C for 24 hours to observe the appearance of purple or blue coloration around each well. This assay was conducted three times for validation.

The synthetic AHLs (Sigma-Aldrich, Saint Louis, USA) used were C6-HSL (N-hexanoyl-DL-homoserine lactone), C8-HSL (N-octanoyl-DL-homoserine lactone), 3-O-C8-HSL (N-3-oxo-octanoyl-DL-homoserine lactone), C10-HSL (N-decanoyl-DL-homoserine lactone), 3-OH-C10-HSL (N-3-hydroxydecanoyl-DL-homoserine lactone), C12-HSL (N-dodecanoyl-DL-homoserine lactone) and 3-O-C12-HSL (N-3-oxo-dodecanoyl-DL-homoserine lactone).

To determine whether the QQ enzyme of B22^T^ was a lactonase type, an acidification assay was carried out (Yates et al., 2002). Briefly, C12-HSL was added to a 24-hour incubation at 28°C. A sterile LB medium supplemented with C12-HSL served as the negative control. Then, the culture and the control were centrifuged, and the pH of the supernatants was adjusted to 2.0 with HCl 1N. Acidified supernatants were then incubated for 24 h at 28°C. The remaining amount of C12-HSL was extracted twice with 1 volume of dichloromethane. Dried extracts were resuspended in acetonitrile and analyzed by HPLC/MS (Torres et al., 2013) as well as by well-diffusion agar-plate assay using *A. tumefaciens* NTL4 (pZLR4) as biosensor strain.

The cellular localization of QQ activity was identified in the supernatant and crude cellular extract (CCE) fractions from a 24 h culture of strain B22^T^ following the methodology previously described. Supernatant and CCE were obtained as described previously and filtered through a 0.22 μm-pore membrane filter (Romero et al., 2014).

To identify genes encoding for QQ activity in strain B22^T^, the whole genome annotated using RAST was used, and potential genes were tested. A protein encoding a predicted acylhomoserine lactone acylase was selected WP_343468083.1 (782 aa) and used to design the specific primers B22-acilgen-Fw: 5’atgtccgggcagttatcgagg-3’ and B22-acilgen-Rev: 5’-ttagtgcgcggccaccctgc-3’. A PCR fragment was amplified and cloned into pGEM-T cloning vector (Promega) following the manufacturer’s instructions. Then, the plasmid construction was transferred into *Escherichia coli* DH5α and the AHL-degradation activity was tested in LB media supplemented with IPTG and AHLs (C10-HSL and C12-HSL) as described above.

### 2.10 Interference of QS systems of plant pathogenic bacteria by *in vitro* co-culture assays

Previous to determine the QQ activity, we determine if the strain B22^T^ interferes the growth of the five plant pathogenic bacteria in an antagonist assay based in a well diffusion method (Frikha-Gargouri et al., 2017). Briefly, 24 h culture of each pathogen was used to prepare an overlay on LB agar plates and 100 μL of aliquots of the filtered supernatant from a 5-day culture of strain B22^T^ were placed on previously prepared wells. After 48 h incubation at 28°C, plates were examined for the appearance of growth inhibition halos surrounding the wells.

QQ activity of strain B22^T^ upon phytopathogenic bacteria was determined by co-culture assays (Torres et al., 2016; Rodríguez et al., 2020). Briefly, 24 h cultures of each pathogen (10^9^ CFU mL^-1^) were co-cultured with 24 h culture of strain B22^T^ (10^9^ CFU mL^-1^) at a ratio of 1:100 in LB medium and incubated for 24 h. As controls, each bacterium was cultured in similar conditions. Then, AHLs from each co-cultures y monocultures were detected using the well-diffusion agar-plate assay as described above, and *C. subtsugae* CV026 and *A. tumefaciens* NTL4 (pZLR4) as bioindicator strains. The abundance of strain B22^T^ and each pathogen in the co-cultures were quantified by serial dilutions and plate counts, using LB medium and LB medium supplemented with 5% (w/v) NaCl and King B agar.

The interference of strain B22 with virulence-associated cellular functions controlled by QS in the pathogens was evaluated by spotting 10 μL of co-cultures and monocultures on different media. Particularly, the following phenotypic characteristics were evaluated: production of acid and alkaline phosphatase (Pikovskaya, 1948; Baird-Parker, 1963); siderophore production (Alexander and Zuberer, 1991); production of hydrolytic enzymes such as amylase and caseinase (Barrow and Feltham, 1993), DNAase (Jeffris et al., 1957), lipases (hydrolysis of Tween 20 and Tween 80) and gelatinase (Tindall et al., 2007).

### 2.11 Virulence assays in potato and carrot slices

The interference of strain B22^T^ with the virulence of *D. solani* LMG 25993^T^ and *P. atrosepticum* CECT 314^T^ was evaluated on potato slices (*Solanum tuberosum*). Infection of *P. carotovorum* subsp. *carotovorum* CECT 225^T^ was tested on potato and carrot (*Daucus carota*) slices while *P. agglomerans* CFBP 11141 was evaluated in cherry tomatoes (*S. lycopersicum* L. var. *cerasiforme*) (Garge and Nerurkar, 2016; Rodríguez et al., 2020). Briefly, vegetables were surface sterilized. Then, potatoes and carrots were cut into slices and placed in Petri dishes while cherry tomatoes were placed entirely with wet filter paper to maintain moisture. The following treatments were conducted for each pathogen: sterilized distillated water, monoculture of pathogen, monoculture of strain B22^T^, pathogen: B22^T^ co-culture (1:100). Each bacterial culture (10^9^ CFU mL^-1^) was centrifuged, resuspended in sterilized distillated water and 15 μL of each condition was inoculated into the tomatoes, carrot and potato slices. Nine replicates of each treatment were conducted, and the assay was repeated three times. After 2–5 days of incubation (depending on each pathogen) at 28°C, maceration areas were calculated by image analysis using ImageJ software (Schneider et al., 2012).

### 2.12 Virulence assays in tomato plants

The impact of AHL degradation of *P. syringae* pv. tomato DC3000 by strain B22^T^ was evaluated in tomato plants (*S. lycopersicum* L. var. Roma) following the technique described elsewhere (Yan et al., 2008). Briefly, tomato seeds were surface-sterilized and sown in pots as described above (section 2.7.). Three pots containing 50 seeds per pot were used for each treatment: sterile distilled water (negative control), strain B22^T^, the pathogen (positive control) and the pathogen: B22^T^ co-culture. The pots were kept in an indoor greenhouse during a long-day photoperiod (16:8h, light:dark) at 25°C and watered with 50 mL sterile distilled water twice a week. After 3 weeks, the pots were exposed to 100% humidity for 16 h to induces stomatal opening and then sprayed with a 5 mL washed cell suspension of *P. syringae* pv. tomato DC3000 (10^9^ CFU mL^-1^), co-culture of pathogen with strain B22^T^ (ratio 1:100), strain B22^T^ (10^9^ CFU mL^-1^) and sterile distilled water. Relative humidity was maintained at 100% for a further 24h to facilitate pathogen infection. Finally, after 4 weeks post-inoculation, affected (necrotic and chlorotic leaves, dead leaves) and unaffected shoot (healthy leaves) were counted and followed by plant harvesting (López-Escudero et al., 2004). The unaffected and affected shoot and roots length total dry weight of 20 plants per treatment were determined.

The chlorophyll fluorescence (Fv/Fm ratio) in tomato leaves was determined using a fluorometer (Handy PEA Hansatech Instruments Ltd.) by measuring the fluorescence on the adaxial side of the leaf for a time of 30 minutes. The parameters of basal fluorescence (F0), maximum fluorescence (Fm), variable fluorescence (Fv), (Fv= Fm-F0), and the ratio (Fv/Fm), an indicator of the functionality of the photochemical conversion of light energy in photosystem II, were obtained (Passari et al., 2019).

### 2.13 Statistical analysis

The data analysis for this study was conducted using GraphPad Prism 9, Statistical Package for the Social Sciences (SPSS) software. The normality of the data was evaluated through either the Shapiro-Wilk test or the D’Agostino and Pearson test. A one-way ANOVA followed by Tukey’s *post hoc* test was employed for comparing group means. A 95% confidence interval was applied in all analyses.

## 3 Results

### 3.1 Phylogenetic and MLSA analyses of strain B22^T^

**Figure 1:**
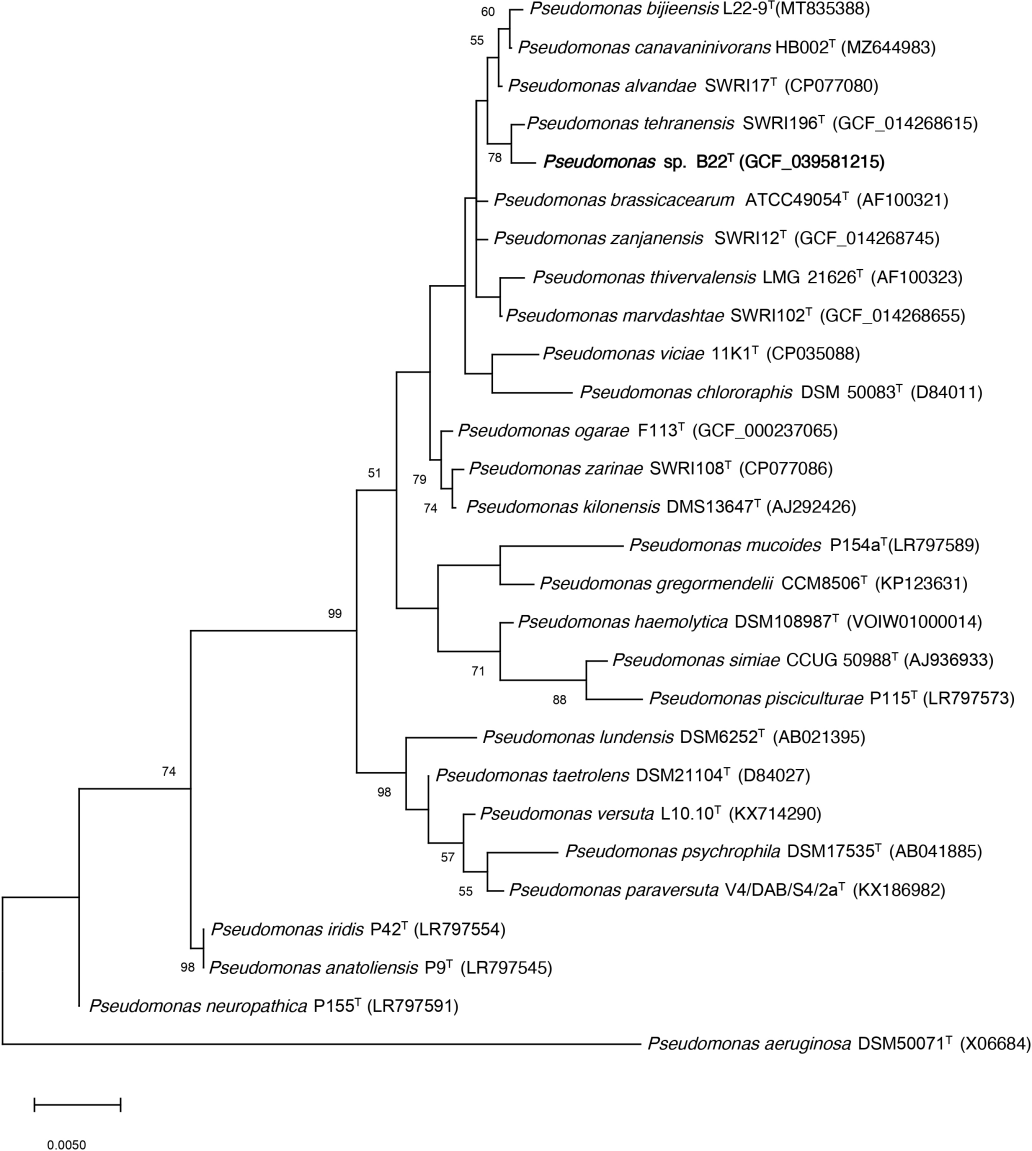
Phylogenetic position of the strain B22^T^ 16S rRNA gene sequence (bold) and its relationship with other related species by using the maximum-likelihood algorithm. The GenBank/EMBL/DDBJ accession number of each sequence is shown in parenthesis. Bootstrap values are expressed as percentages of 1,000 replications, and those >50% are shown at branch points. Bar shows sequence divergence. Bar−0.005 substitutions per nucleotide position. *P. aeruginosa* DSM50071^T^ sequence was used as an outgroup.

Strain B22^T^ was isolated from the rhizospheric soil of the halophyte *Salicornia hispanica.* The almost complete 16 rRNA gene sequence ( ~1,500 bp) of strain B22^T^ was obtained by PCR, which was identical to that extracted from its genomic sequence. Strain B22^T^ showed the highest sequence identity to *Pseudomonas tehranensis* SWRI 196^T^ (99.86%), *P. alvandae* SWRI 17^T^ (99,66%) and *P. canavaninivorans* HB 002^T^ (99,59%), while identities below 97% were obtained with other species from *Pseudomonas* genus. Phylogenetic analysis of its 16S rRNA gene sequences and other related strains through a phylogenetic tree reconstruction using the maximum-likelihood algorithm showed that this strain is a member of the genus *Pseudomonas* and forms a cluster with the *P. tehranensis* species, which showed the highest sequence similarity (**Figure 1**). A similar phylogenetic distribution was obtained when the neighbor-joining and maximum parsimony algorithms were used (**Supplementary Figure S1**).

The MLSA resulting Neighbor-joining tree, obtained by concatenating the sequences of four housekeeping genes (*16S rRNA, gyrB, rpoD*, and *rpoB*) also indicated that B22^T^ and *Pseudomonas tehranensis* SWRI196^T^ forms a cluster with a bootstrap value of 100% (**Supplementary Figure S2**).

### 3.2 Whole-genome sequencing and assembly

The draft genome of strain B22^T^ was manually curated, resulting in a genome size of over 5.7 Mbp across 32 contigs. The assembly quality was evaluated using the Quality Assessment Tool for Genome Assemblies (QUAST), yielding an N50 value of 237,4 kb, an L50 of 9, and approximately 300X coverage, indicating high-quality sequencing. Annotation of the draft genome using PGAP (Tatusova et al., 2016) identified 5014 protein-coding genes (PCGs). This genome sequence was deposited in the GenBank/EMBL/DDBJ database under accession number JBCNTJ010000000 and was used for further analysis.

### 3.3 *In silico* G+C content, ANI and DDH calculations

The *in silico* analysis of the G+C content in the draft genome of strain B22^T^ revealed a value of 60.5 mol%. Average nucleotide identity (ANI) values, calculated using OrthoANI, yielded a similarity between strain B22^T^ and *P. tehranensis* SWRI 196^T^ of 95.61%, within the limit of the species delimitation threshold of 95–96% (Chun et al., 2018). The values for strain B22^T^ and other related species were below this threshold (**Supplementary Table S1**).

Digital DNA-DNA hybridization (dDDH) analysis of the whole-genome sequences of strain B22^T^ and closely related species also produced values below the species delineation threshold (70%) (**Supplementary Table S1**).

### 3.4 Phylogenetic analysis of core orthologous proteins

**Figure 2:**
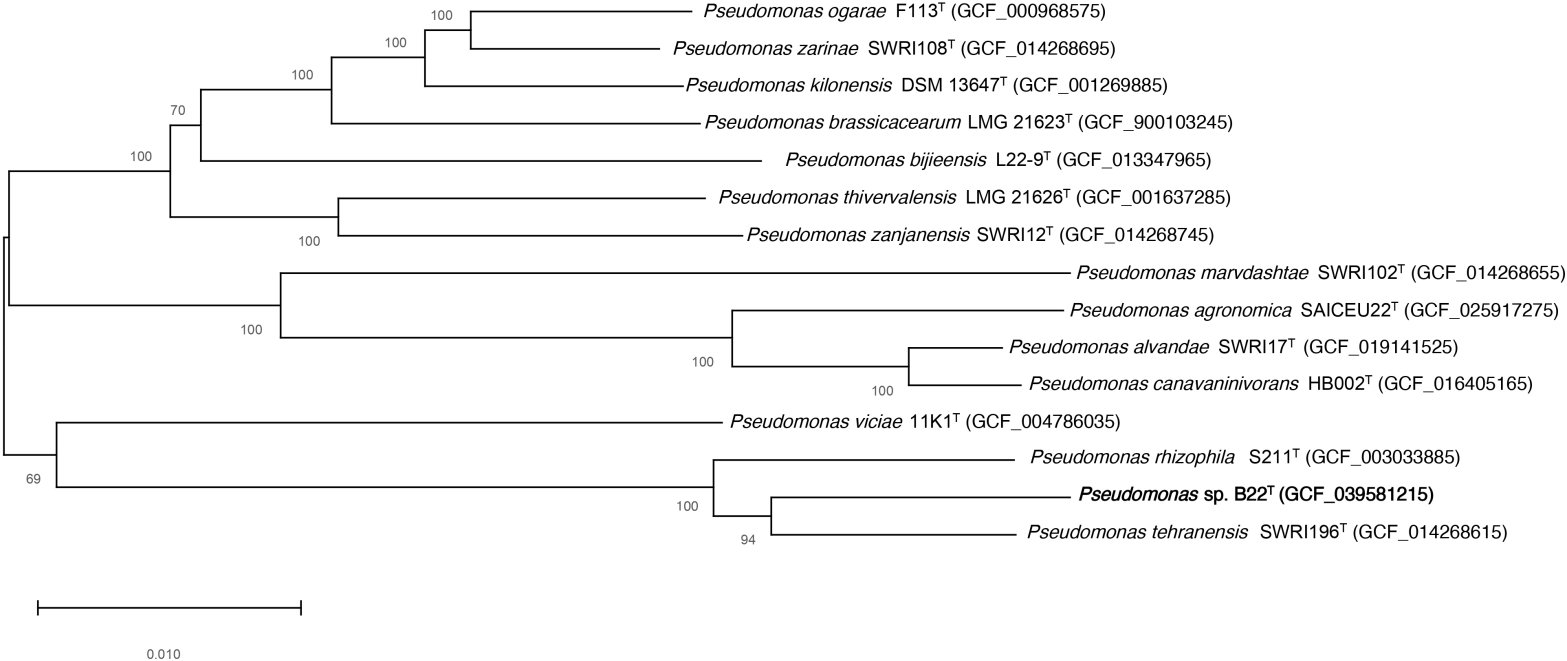
Tree constructed according to the maximum-likelihood method based on 3093 core orthologous proteins of the strain B22^T^ (bold) and the available genomes of *Pseudomonas* related species. Bootstrap values are expressed as percentages of 1,000 replications, and those over 50% are shown at branch points. Bar−0.010 substitutions per nucleotide position.

The concatenated alignment of 3093 core orthologous proteins from strain B22^T^ and related species of the genus *Pseudomonas*, was used to construct a maximum-likelihood phylogenetic tree. The resulting tree is shown in **Figure 2**.

### 3.5 Chemotaxonomic characteristics

The predominant cellular fatty acids of the strain B22^T^ were summed feature 3 (contains C_16:1_ ω7*c* and/or C_16:1_ ω6*c*) (41.07%), C_16:0_ (28.87%), and summed feature 8 (contains C_18:1_ ω7*c* and C_18:1_ ω6*c*) (12.04%). The major polar lipids of strain B22^T^ were diphosphatidylglycerol and phosphatidylethanolamine. Other polar lipids present were phosphatidylglycerol, an unidentified glycolipid, an unidentified phospholipid and an unidentified lipid (**Supplementary Figure S3**). The predominant respiratory quinone of strain B22^T^ was ubiquinone-9 (Q-9).

### 3.6 Morphological, physiological and biochemical characteristics

Cells of strain B22^T^ were Gram-negative, rod-shape and motile (**Supplementary Figure S4**). It forms creamy small colonies after 48 h of incubation in LB agar plates. Growth was positive on Simmons citrate agar and fluorescent colonies were detected on King B agar. Strain B22^T^ did not produce endospores. It was catalase and oxidase positive. It was aerobe, with growth temperatures ranging from 4 to 40°C and with an optimum temperature at 28°C. It grew in pH range from 5 to 10 with pH 7 as optimum. Strain B22^T^ resulted to be halotolerant as it grew from 0 to 5% (w/v) of NaCl, with 0.5-2.5% (w/v) being the optimum concentration. The differential characteristics of this strain with respect to the most closely related species, *P. tehranensis* SWRI 196^T^, *P. alvandae* SWRI 17^T^ and *P. canavaninivorans* HB 002^T^ were listed in **Table 1**. For example, strain B22^T^ doesn’t assimilate D-trehalose, D-serine, D-sorbitol, ß-hydroxy-phenylacetic acid while *P. tehranensis* SWRI 196^T^ does. B22^T^ is sensitive to fusidic acid while *P. tehranensis* SWRI 196^T^ is resistant. More phenotypical characteristics of strain B22^T^ were detailed in the species description and in **Supplementary Tables S2**, **S3**.

**Table 1:** Differential characteristic between strain B22^T^ with respect to its closest species of the *Pseudomonas* genus.

Characteristic	1	2	3	4
API 20NE tests				
Protease	+	+	–	–
D-mannose assimilation	+	w	w	–
Oxidation of (API 50 CH)				
Glycerol	–	–	+	–
D-arabinose	–	–	+	–
D-ribose	–	–	+	–
D-xylose	–	–	+	–
L-xylose	–	–	+	–
D-galactose	–	–	+	–
D-fructose	–	–	+	–
D-mannose	–	–	+	–
Inositol	–	–	+	–
D-mannitol	–	–	+	–
D-sorbitol	–	–	+	–
D-melibiose	–	–	+	–
D-trehalose	–	+	+	–
gentiobiose	–	–	+	–
D-fucose	–	–	+	–
L-arabitol	–	–	+	–
Potassium gluconate	–	–	+	+
Enzymes (API ZYM)				
Esterase (C8)	+	+	+	w
Acid phosphatase	w	w	–	–
Napthol-AS-BI-phosphohydrolase	w	+	+	–
α−fucosidase	w	w	w	–
Assimilation of (Biolog)				
Dextrina	w	w	–	w
D-trehalose	+	+	+	w
D-melibiose	w	–	–	–
N-acetyl-D-glucosamine	w	+	w	+
N-acetyl-D-mannosamine	–	–	w	w
3-methyl glucose	w	–	–	w
D-fucose	+	w	w	w
L-fucose	w	w	–	w
L-rhamnose	w	w	–	–
D-serine	–	+	w	–
D-sorbitol	–	+	+	+
α-arabitol	w	+	+	+
*myo*-inositol	w	+	+	+
D-glucose-6-PO_4_	w	–	–	–
D-aspartic Acid	–	–	+	+
Glycyl-L-proline	–	–	–	w
L-arginine	w	+	+	+
L-glutamic acid	w	+	+	+
L-histidine	+	+	w	+
L-serine	+	+	w	+
Pectin	+	+	–	+
Glucuronamide	+	+	w	+
β-hydroxy-phenylacetic acid	–	+	–	–
α-keto-glutaric acid	w	+	w	+
D-malic acid	–	–	–	+
Bromo-succinic acid	+	+	w	+
Tween 40	w	+	–	+
Acetoacteic acid	–	–	w	w
Formic acid	w	w	w	+
Sensitivity to (Biolog)				
Nalidixic acid	+	+	–	+
Aztreonam	+	+	w	+
Fusidic acid	–	+	+	+
DNA G + C content (*in silico*) (mol%)	60.5	60.5	61	61
Main Quinone	Q9	Q9	Q9	Q9

Strains: 1, B22^T^; 2, *P. tehranensis* SWRI 196^T^; 3, *P. alvandae* SWRI 17^T^; 4, *P. canavaninivorans* HB 002^T^. All data were obtained from this study. Only the most relevant results are shown. The complete results can be found in **Supplementary Table S2**. For assimilation and oxidation of carbon compounds: +, positive; -, negative; w, weakly positive. For sensitivity: +, insensitive (can grow in its presence); -, sensitive (cannot grow in is presence).

Concerning the PGP activity, strain B22^T^ resulted positive for the hydrolysis of Tween 20 and Tween 80 as well as for phytase and acid phosphatase. It also produced IAA and siderophores. Nevertheless, it did not produce amylase, cellulase, caseinase, lecithinase, alkaline phosphatase and chitinase (**Supplementary Table S3**).

### 3.7 Growth promotion and colonization assays

**Figure 3:**
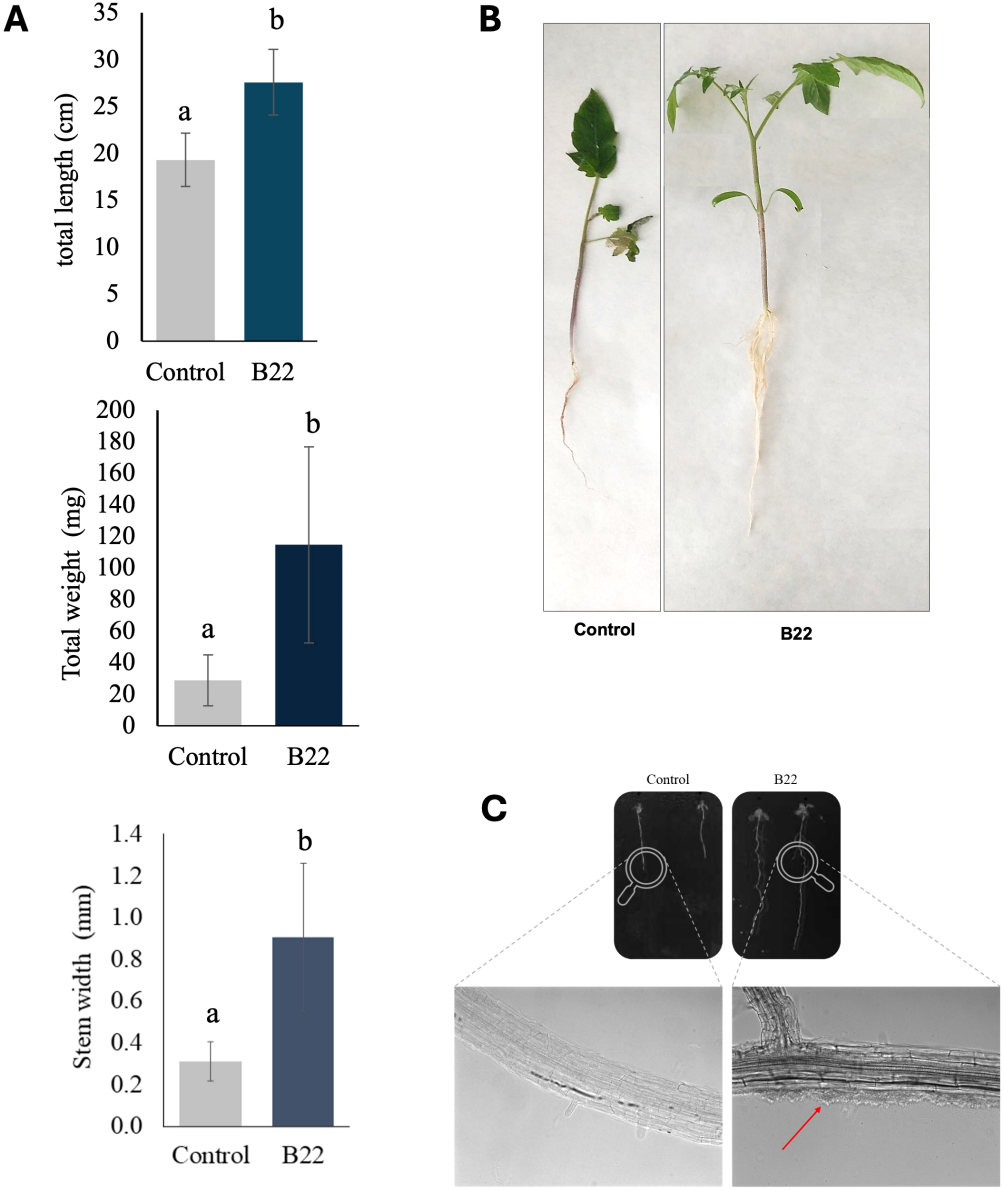
Plant growth-promoting assay of B22^T^. **(A)** Effects on plant height, dry weight and stem diameter of tomato plants. Values are expressed as mean ± standard deviation. Data presented are means ± SE (n = 12). Different letters indicate significant differences according to Tukey’s test (p < 0.05). **(B)** Photographs of the tomato plants. **(C)** Microscopy observation of the colonization of *A*. *thaliana* roots by strain B22^T^. Differential interference contrast (DIC) micrographs taken at 200X.

To assess the PGP activity of the halotolerant bacteria in tomato plants 28 days post-inoculation, the plants’ morphological characteristics were evaluated. Plant dry weight and length were significantly higher compared to non-inoculated plants 114.73 ± 62.07 mg *vs* 19.34 ± 2.83 cm and 27.6 ± 3.51 cm *vs* 19.34 ± 2.83 cm, respectively (**Figures 3A, B**). A similar pattern was observed for stem diameter, with plants inoculated with the B22^T^ strain showing the largest stem diameters compared to the non-inoculated plants 0.9 ± 0.35 mm *vs* 0.31 ± 0.1 mm, respectively.

The colonization of plant roots was studied in *Arabidopsis thaliana* through microscopy. The micrographs obtained by differential interference contrast showed small bacterial masses at the base of the root hairs of plants treated with the strain B22^T^ strain, and the absence of bacteria in the control roots (**Figure 3C**).

### 3.8 Characterization of the AHL degradation activity of strain B22^T^

The ability of AHL degradation of strain B22^T^ was tested against a wide range of synthetic AHLs through a well-diffusion agar-plate assay. Strain B22^T^ was able to completely degrade the following AHLs: C6-HSL, C10-HSL, 3-OH-C10-HSL, C12-HSL and 3-oxo-C12-HSL as the corresponding biosensor strains (*C. subtsugae* CV026, *C. violaceum* VIR07 and *A. tumefaciens* NTL4 pZLR4) were not activated. Nevertheless, C8-HSL and 3-oxo-C8-HSL were not degraded (**Supplementary Figure S5**).

To determine whether the enzymatic activity of strain B22^T^ was due to a AHL lactonase type, an acidification assay to test the lactone ring closure was conducted using C12-HSL, an AHL totally degraded by this strain. The remaining AHLs in the acidified cell-free supernatant (pH 2) as well as in the control (cell-free supernatant at pH 7) were detected by a well-diffusion agar-plate assay using *A. tumefaciens* NTL4 (pLR4) as biosensor strain. The AHL concentration was not restored in the acid conditions. This result was confirmed by HPLC-MRM, suggesting that QQ activity in this strain was not caused by an AHL lactonase (**Supplementary Figure S6A**).

The cellular localization of the enzyme was assessed by testing C10-HSL and C12-HSL degrading activity of strain B22^T^ in cell-free supernatant (SN) and crude cellular extract (CCE). Well-diffusion agar-plate assay with *C. violaceum* VIR07 and *A. tumefaciens* NTL4 (pZLR4) showed that QQ activity was detected in SN, whilst no AHL degradation was seen in CCE, suggesting that the enzyme was secreted (**Supplementary Figure S6B**).

To identify the gene responsible for AHL-degradation activity in strain B22^T^, the automatic annotation of the whole genome was used to select a gene that encodes potential acylase protein WP_343468083.1 (782 aa). A DNA fragment of ~2349 bp was amplified by PCR, using designed specific primers, purified and cloned in the pGEM-T vector and then, expressed in *E. coli* DH5α. The QQ activity of the plasmid construction pGEMT-B22 against C10-HSL and C12-HSL was confirmed by the well-diffusion agar-plate assay (**Supplementary Figure S6C**). In addition, BLASTp analysis revealed that the predicted protein encoded by the identified gene WP_343468083.1 (782 aa) shared 95.27% and 93.48% identity with the acylase sequences from *P. fluorescens* and *P. tehranensis*, respectively, both of which are annotated as members of the penicillin acylase family.

### 3.9 Interference of bacterial phytopathogen QS systems and impact on associated phenotypes by strain B22^T^

The QQ activity of strain B22^T^ was tested against the AHLs produced by *D. solani* LMG 25993^T^, *P. atrosepticum* CECT 314^T^, *P. carotovorum* subsp. *carotovorum* CECT 225^T^, *P. agglomerans* CFBP 11141 and *P. syringae* pv. tomato DC3000 to evaluate its potential use for biocontrol. In these pathogens, AHLs have been characterized and correlated to the expression of virulence factors. Firstly, an antagonism assay was conducted to evaluate whether strain B22^T^ interferes with the growth of each pathogen and no inhibitory effect was detected (data not shown). The five plant pathogens were co-cultured with strain B22^T^ in LB medium at a ratio of 1:100 for 24 h. Monocultures of each bacterium were used in each case as controls. The concentration of each bacterium was determined by counting plate using selective media and they remained stable through the experiment. Then, AHLs were detected by well-diffusion agar-plate assay with *A. tumefaciens* NTL4 (pLRZ4) and *C. subtsugae* CV026 as bioindicator strains. The results indicated that under our assay conditions, strain B22^T^ degraded almost completely the AHLs produced by *D. solani* LMG 25993^T^ and *P. agglomerans* CFBP 11141 as no activation of the biosensors were visualized in their respectively co-cultures. In respect of co-cultures of strain B22^T^ and the pathogens *P. atrosepticum* CECT 314^T^, *P. carotovorum* subsp. *carotovorum* CECT 225^T^ and *P. syringae* pv. tomato DC3000, a partial AHL degradation was observed (**Supplementary Figure S7A**).

Based on the capacity of strain B22^T^ to degrade AHLs produced by the tested phytopathogens, similar co-cultures were conducted and used to evaluate the effect on the production of cellular functions regulated by QS in each pathogen. Since strain B22^T^ produced numerous PGP traits, the possible reduction of some phenotypic characteristics in the pathogens could not detected (**Supplementary Table S3**). As results, some phenotypes of pathogens were inhibited when co-cultured with strain B22^T^. The production of alkaline phosphatase and lecithinase in *D. solani*; the production of Tween 20 in *P. atrosepticum* and the production of amylase in *P. agglomerans* was inhibited in the presence of strain B22^T^ while a reduction in DNAase production was observed in *P. carotovorum.* Regarding *P. syringae* pv. tomato, no traits were found to be affected in our phenotypic assays (**Supplementary Figure 7B**).

### 3.10 Bioassay of strain B22^T^ in controlling phytopathogens on vegetables and fruits and tomato plants

**Figure 4:**
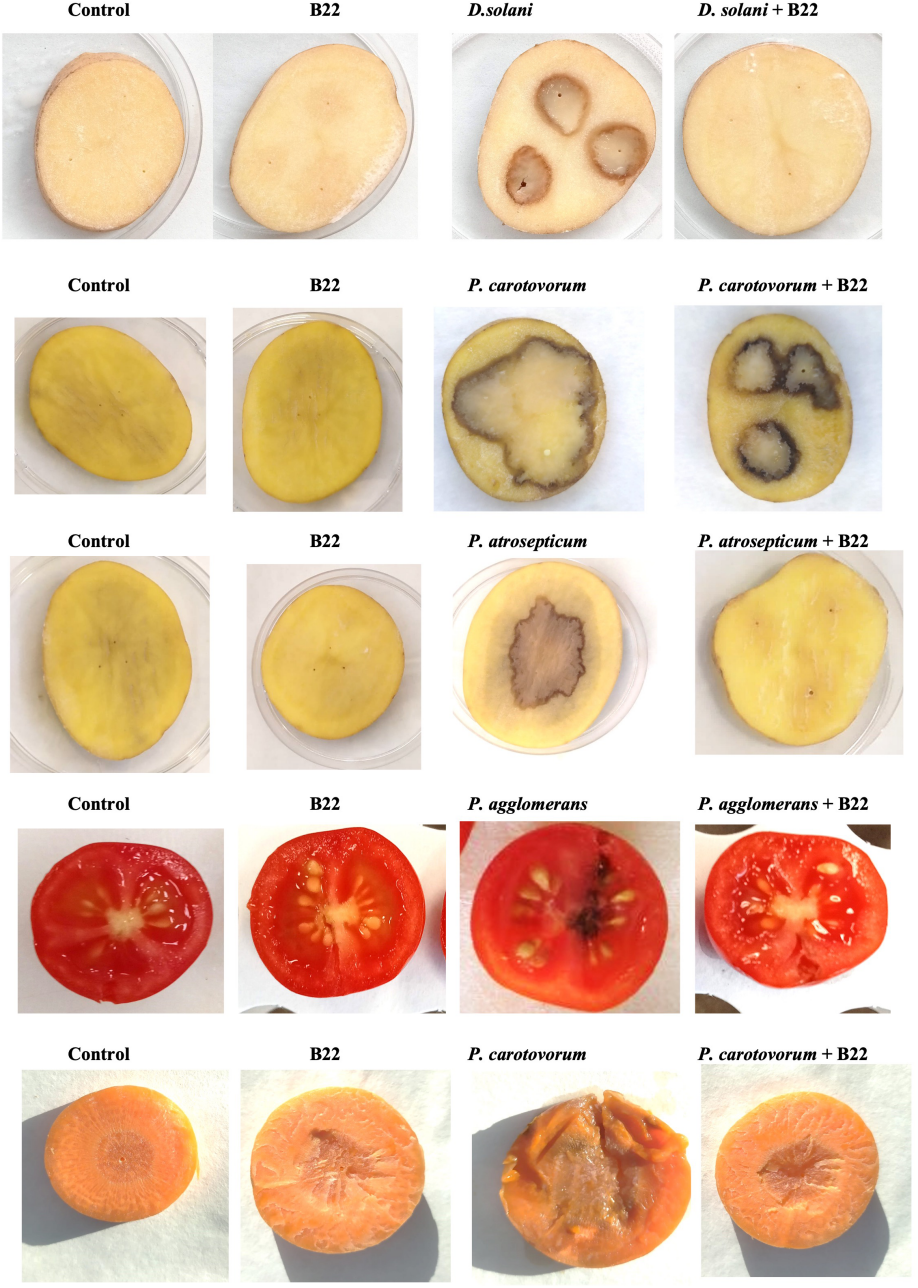
Virulence assay in potato tuber, carrot slices and cherry tomatoes. Assessment of virulence and maceration of cultures and co-cultures of strain B22^T^ and different pathogens inoculated on the surface of potato and carrot slices after 2 days of incubation and cherry tomatoes after 5 days of incubation. Sterile water was used as negative control.

To evaluate whether the AHL degradation produced by strain B22^T^ in co-culture with the plant pathogens has an impact on their *in vivo* virulence, different assays on fruits and plants were conducted. Co-cultures of strain B22^T^ with the pathogens were prepared as described above. Additionally, sterile water and monoculture of each strain were used as controls. Mono and co-cultures of *D. solani*, *P. atrosepticum* and *P. carotovorum* were inoculated on the surface of potato slices. In the case of *P. carotovorum*, cultures were also inoculated on the surface of carrot slices. Mono and co-cultures of B22^T^ with *P. agglomerans* were inoculated in cherry tomatoes. As it is shown in **Figure 4** and **Supplementary Figure S8**, under our assay conditions, strain B22^T^ drastically reduced the virulence capacity of *D. solani* and *P. atrosepticum* to produce soft rot due to maceration area of 5.46 ± 1.79% and 5.22 ± 0.52% respectively, while a maceration area of 65.38 ± 3.21% and 14.96 ± 1.25% was originated by each pathogen in monoculture respectively. Regarding *P. carotovorum*, strain B22^T^ significantly diminished the capacity of the pathogen to induce soft rot in slice carrots (7.06 ± 0.58%) and potatoes (18.19 ± 0.06%) as compared to the 46.17 ± 3.12% and 34.44 ± 8.40% observed by the pathogen in monoculture respectively. In the case of *P. agglomerans*, cherry tomatoes inoculated with the pathogen exhibited tissue damage (25 ± 0.01%) while a significant reduction of the infection symptoms were observed in the presence of strain B22^T^ (2.5 ± 0.01%). In all of assays conducted with the different types of vegetables, the inoculation of strain B22^T^ did not originate any infection symptoms.

**Figure 5:**
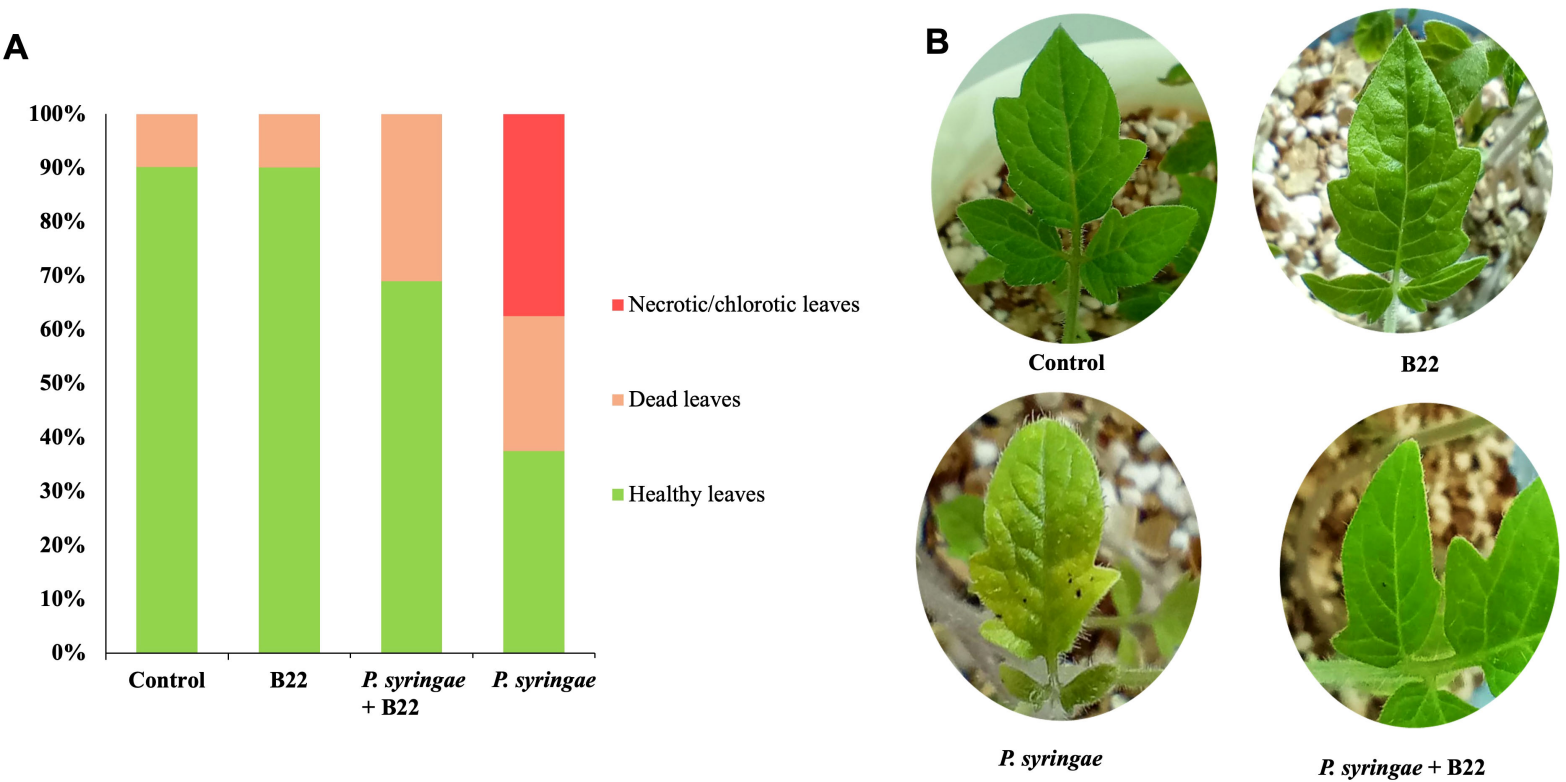
Infection assay in tomato plants treated with sterile water (control), strain B22^T^ and *Pseudomonas syringae* pv*. tomato* DC3000 mono-cultures and phytopathogen- B22^T^ co-culture. **(A)** Total percentage of healthy, necrotic/chlorotic and dead leaves after each treatment. **(B)**. Photograps showing the necrotic symtoms on leaves after treatment.

The use of strain B22^T^ as biocontrol agent was also evaluate by conducting *in vivo* assays of tomato plants infected with *P. syringae* pv. *tomato* DC3000. As it is shown in **Figure 5**, tomato plants treated with the pathogen:strain B22^T^ co-culture showed less infection symptoms than those infected with the pathogen alone. Indeed, tomato plants treated with the co-culture showed a significative increase of 31.49% in healthy leaves as compared to plants inoculated with the pathogen. Moreover, the number of necrotic/chlorotic leaves in plants treated with the co-culture was 0 while the value was 37.5% in those plants treated with the pathogen alone. Negative control and strain B22^T^-treated plants showed some damaged leaves associated with natural senescence.

Regarding fluorescence parameters, plants exposed to the monoculture of B22^T^ and the pathogen:strain B22^T^ co-culture showed an increase of the optimum quantum yield of PSII, represented by the Fv/Fm ratio, compared with plants inoculated with the pathogen (7 and 15% respectively) (data not shown). These results indicated the benefits of the strain B22^T^ that clearly attenuated the virulence of the pathogen.

## 4 Discussion

Bacterial plant diseases represent a major threat to agricultural productivity, causing severe economic losses and compromising the quality and safety of crops. To address this challenge, sustainable strategies are being developed to replace or minimize the use of chemical treatments, which often contribute to the emergence of resistant bacterial strains. One promising approach involves the use of microorganisms as biostimulants and biocontrol agents (Sharma et al., 2022; Chaudhary et al., 2024). In this context, among biocontrol mechanisms, as QS regulates numerous cellular functions, including virulence factors, the enzymatic degradation of AHL signal molecules in phytopathogenic bacteria has emerged as a promising and sustainable strategy to combat bacterial infection (Grandclément et al., 2016; Hartmann et al., 2021; Zhu et al., 2023).

In this study, we taxonomically identified the strain B22^T^, which was isolated from the rhizospheric soil of *Salicornia hispanica* and evaluated its PGP and QQ properties to control bacterial plant infections.

In respect of the genomic analyses, the *in silico* analysis of the G+C content revealed a value of 60.5 mol%, which falls within the reported range average for the *Pseudomonas* genus (61.2 mol%) (Hesse et al., 2018). The OrthoANI similarity value between strain B22^T^ and *P. tehranensis* SWRI 196^T^ was 95.61%, placing it within the species delimitation threshold of 95–96% (Richter and Rosselló-Móra, 2009; Chun et al., 2018). The digital DNA-DNA hybridization values between strain B22^T^ and its closely relatives were clearly bellow of the 70% species delineation threshold (Stackebrandt and Goebel, 1994), reinforcing its classification as a novel species. Phylogenetic analysis based on the concatenated alignment of 3,093 core orthologous proteins from strain B22^T^ and related *Pseudomonas* species further supports the propose taxonomic placement.

Regarding to the cellular fatty acids profile and the predominant respiratory quinone of strain B22^T^ were similar to those of its closest related species within the *Pseudomonas* genus. Among phenotypic tests performed, slight differences were observed between strain B22^T^ and the closest relatives as expected since they belong to the *P. corrugata* subgroup (Poli et al., 2024).

Taken together, genomic, phylogenetic and chemotaxonomic analyses strongly indicate that strain B22^T^ represents a novel species, for which the name *Pseudomonas halotolerans* is proposed.

Strain B22^T^ belongs to a genus in which certain species such as *P. fluorescens, P. chlororaphis, P. protegens, P. putida* and *P. segetis* have demonstrated considerable potential as PGP and biocontrol agents against plant diseases. They can efficiently colonize the rhizosphere and produce beneficial effects through various mechanisms, including the production of bioactive compounds such as hormones, enzymes, antibiotics and siderophores, as well as the induction of systemic resistance in plants (Vejan et al., 2016; Gouda et al., 2018; Arrebola et al., 2019; Rodríguez et al., 2020; Raio and Puopolo, 2021; Al-Karablieh et al., 2022; Bakaeva et al., 2022; Ajijah et al., 2023).

We assessed the PGP properties of strain B22^T^ through an *in vivo* experiment with tomato plants under sterile conditions. Our findings revealed that strain B22^T^ significantly enhanced all measured growth parameters in tomato plants compared to the control group (increase of 30%, 74.8% and 65.5% in total length, weight and stem width). Notably, plants treated with strain B22^T^ exhibited a remarkable increase in root length and weight, highlighting its strong root growth stimulation activity. These results could be due to several traits linked to its ability to promote plant growth identified in strain B22^T^, including phosphate solubilization (through acid and phytase activities), and the production of IAA, siderophores, and enzymes. These biochemical activities enhance plant nutrition as reported in other bacteria (Lavakush et al., 2014; Prasad et al., 2015). In addition, as strain B22^T^ was isolated from the rhizosphere, its ability to colonize plant roots was confirmed using the model organism *A. thaliana*, reinforcing its potential as an effective root-associated growth-promoting agent.

The plant-growth promotion on tomato plants has been evaluated in several *Pseudomonas* species by other authors. However, no studies have been conducted on the most phylogenetically related species needed for comparison with our data. It has been reported that the inoculation with *P. geniculata* in tomato plants increased aerial and root biomass by 7 and 9%, respectively (Gopalakrishnan et al., 2015), while *P. fluorescens* enhanced aerial dry weight by 4.7% (Siddiqui et al., 2001). Notably, inoculation with *P. segetis* led to significant increases in aerial and root dry weight (19.28% and 21.54%, respectively), although no substantial differences were observed in shoot and root length of tomato plants (Rodríguez et al., 2020).

The QQ activity of strain B22^T^ exhibited versatility for a wide range of AHLs (C6-C12-HSL), with or without different chemical substitutions, except for C8-HSL and 3-oxo-C8-HSL. The enzymatic mechanism responsible for this QQ activity was not attributed to an AHL lactonase but was more likely mediated by another type of enzyme, such as an acylase or oxidoreductase. The heterologous expression of a putative QQ enzyme-encoding gene identified in the annotated genome of strain B22^T^, combined with BLASTp analysis of the predicted protein, strongly support the hypothesis that the QQ enzyme from strain B22^T^ belong to the acylase family.

AHL acylases has been identified more frequently than lactonases in other *Pseudomonas* species such as PvdQ (Bokhove et al., 2010) and QuiP (Huang et al., 2003) from *P. aeruginosa*; HacA and HacB from *P. syringae* (Shepherd and Lindow, 2009); acylase from *P. segetis* P6 (Rodríguez et al., 2020), *P. multiresinivorans* QL-9a (Liu et al., 2023) and *Pseudomonas* sp. HS-18 (Wang et al., 2022). Studies have shown that AHL acylases exhibit greater substrate specificity than lactonases and degrade long-chain AHLs more efficiently than those with short side chains (Yu et al., 2018). However, strain B22^T^ has demonstrated to possess a wide AHL degradation profile.

AHL-degrading enzymes have been widely studied due to their ability to degrade AHL molecules through catalytic processes without the need to penetrate bacterial cells. This characteristic makes them less invasive than QSIs while potentially exhibiting bactericidal effects (Bzdrenga et al., 2017). Additionally, it has been suggested that plant growth-promoting bacteria may enhance their competitiveness in root colonization and contribute to the biocontrol of AHL-dependent plant pathogens through their AHL-degrading activity (Hartmann et al., 2021).

Since strain B22^T^ exhibited significant QQ activity against a wide range of synthetic AHLs, we evaluated its ability to degrade AHLs produced by plant bacterial pathogen in co-cultures to assess its potential as biocontrol agent in agriculture. Our results showed that strain B22^T^ completely degraded AHLs produced by *D. solani* and *P. agglomerans*, while partial degradation was observed in the co-cultures with *P. atrosepticum*, *P. carotovorum subsp. carotovorum* and *P. syringae* pv. tomato DC3000. In addition to AHL degradation, the suppression of QS-regulated phenotypes further supports the QQ activity of strain B22^T^. In our co-culture experiments, the impact of AHL degradation on the production of some enzymatic activities in plant pathogens could not be tested, as strain B22^T^ itself produces several hydrolytic enzymes. However, we observed a significant reduction in the production of alkaline phosphatase and lecithinase in *D. solani*, reduction of Tween 20 hydrolysis in *P. atrosepticum* and the suppression of amylase production in *P. agglomerans* was significantly reduced in the presence of strain B22^T^. The observed impact of these phenotypes is in concordance with previous studies demonstrating the role of QS in controlling virulence factors, including the production of hydrolytic enzymes in plant pathogens (Faure and Dessaux, 2007; Helman and Chernin, 2015; Garge and Nerurkar, 2016; Reina et al., 2019).

Moreover, to support the potential of strain B22^T^ as a biocontrol agent, vegetable infection assays demonstrated its ability to attenuate the virulence of multiple plant pathogens. The addition of strain B22^T^ significantly reduced soft rot development caused by *D. solani* (91.64%) and *P. atrosepticum* (65%). Similarly, the virulence of *P. carotovorum* was markedly reduced in both potato (45.6%) and carrot (85.5%) slices. Notably, results obtained with *P. agglomerans* (60%) in cherry tomatoes indicate that strain B22^T^ can mitigate tissue damage beyond typical soft rot pathogens, suggesting broader biocontrol potential. These results are according to previous studies reporting the use of AHL-degrading bacteria to attenuate phytopathogen virulence (Vega et al., 2020; Rodríguez et al., 2020; Liu et al., 2023; Roca et al., 2024). It has been shown that the production of cell-wall degrading enzymes (PCWDE) promotes the destruction of plant tissue and is in many bacteria regulated by AHLs (Garge and Nerurkar, 2016).

*In vivo* assays on tomato plants infected with *P. syringae* pv. tomato DC3000 provides further evidence of strain B22^T^’s protective role. Plants treated with the co-culture containing strain B22^T^ exhibited a significant increase in healthy leaves and a complete absence of necrotic or chlorotic symptoms compared with those exposed to the pathogen alone, highlight its effectiveness in reducing disease severity. This finding aligns with previous studies demonstrating the use of QQ bacteria to mitigate foliar pathogen virulence (Vega et al., 2020). Photosynthesis is one of the most important processes affected by the infection with bacterial pathogens and photosystem II (PSII) may play a significant role in the plant’s defense (Zou et al., 2005). Previous studies observed the decrease of the maximum quantum efficiency of PSII (Fv/Fm) in plants treated with *P. syringae* compared to uninfected control plants (Bonfig et al., 2006; Rodríguez-Moreno et al., 2008). In this study the treatment of tomato plants with the monoculture of B22^T^ and the pathogen:strain B22^T^ co-culture increased significantly the ratio Fv/Fm compared to the plants treated with the phytopathogen. Similar results were found previously in tomato plants inoculated with the actinobacteria *Streptomyces thermocarboxydus* as a biocontrol agent to control *Fusarium* wilt disease (Passari et al., 2019). These results suggest that strain B22^T^ not only promotes tomato plants growth but may also enhances plant defense responses, further limiting pathogen impact.

This significant reduction in virulence observed in our assays suggests that strain B22^T^ may interfere with the pathogenicity mechanisms of these bacteria, potentially through AHL degradation and other mechanisms that need to be elucidated. Notably, strain B22^T^ did not inhibit pathogen growth, as bacterial concentrations remained stable throughout the experiment. However, the biocontrol mechanisms in certain *Pseudomonas* spp., have been linked to the production of antibiotics, siderophores and volatile organic compounds (Arseneault et al., 2016; Raio and Puopolo, 2021).

Previous studies have shown that plant-associated bacteria can attenuate pathogen induced QS-dependent plant infections by degrading AHL molecules (Vega et al., 2020; Rodríguez et al., 2020; Liu et al, 2023; Roca et al., 2024). Notably, the heterologous expression of QQ enzymes in bacterial pathogens, such as *P. carotovorum* subsp. *carotovorum* (Crépin et al., 2012; Torres et al., 2017; Dong et al., 2000), *D. chrysanthemi* (Hosseinzadeh et al., 2017) and *Burkholderia cenocepacia* (Wang et al., 2022), has been found to drastically reduce AHL accumulation and virulence.

An important observation from this study is that strain B22^T^ did not cause any visible infection symptoms in any tested vegetables and plants, highlighting its safety as a potential biocontrol agent. This aspect is crucial for agricultural applications, ensuring that introducing strain B22^T^ does not pose risks of pathogenicity to crops. To date, although QQ activity has been identified in certain *Pseudomonas* strains (Huang et al., 2003; Shepherd and Lindow, 2009; Bokhove et al., 2010; Wang et al., 2022; Liu et al., 2023) but relative few studies have described PGP strains of *Pseudomonas* capable of degrading AHL signal molecules produced by bacterial plant pathogens (Rodríguez et al., 2020).

Overall, our results demonstrate that strain B22^T^ exhibits a broad-spectrum QQ activity, mediated by a secreted enzyme distinct from AHL lactonases. The potential biocontrol application of strain B22^T^ is further supported by its ability to degrade AHLs without negatively affecting pathogen growth. This characteristic makes it a promising candidate for sustainable plant protection strategies that target QS without exerting selective pressure on bacterial populations, which could otherwise lead to resistance development.

### 4.1 Description of *Pseudomonas halotolerans* sp. nov.

*Pseudomonas halotolerans* (ha.lo.to′le.rans. Gr. n. *hals* salt; L. pres. part. *tolerans* tolerating; N.L. part. adj. *halotolerans* referring to the ability of the organism to tolerate high salt concentrations).

Cells of this species are aerobic, Gram-negative, non-sporeforming, motile and rod-shaped. In LB agar plates, cells can grow forming creamy and small colonies. Growth occurs in 0-5% (w/v) NaCl (optimum 0.5-2.5%), at pH varying from pH 5 to 10 (optimum pH 7), and within the temperature range of 4-40° C (optimum 28° C). Catalase and oxidase tests are both positive. Results obtained with Biolog GEN III microplates indicate that cells can use the following substrates as carbon and energy sources: D-trehalose, sucrose, α-D-glucose, D-mannose, D-fructose, D-galactose, D-fucose, inosine, D-mannitol, glycerol, D-fructose 6-PO_4_, L-alanine, L-aspartic acid, L-histidine, L-pyroglutamic acid, L-serine, pectin, D-galacturonic acid, L-galacturonic acid lactone, D-gluconic acid, D-glucuronic acid, glucuronamide, mucic acid, quinic acid, D-saccharic acid, methyl pyruvate, L-lactic acid, citric acid, L-malic acid, bromo-succinic acid, gamma-amino-butyric acid β-hydroxyl-D L-butyric acid, propionic acid and acetic acid. All other substrates in the Biolog GEN III panel did not or only weakly support cell growth. Based on Biolog Gen III sensitivity assays, cells of this species can grow at 1% w/v sodium lactate, and are resistant to troleandomycin, rifamycin SV, lincomycin, guanidine HCl, niaproof 4, vancomycin, tetrazolium violet, tetrazolium blue, lithium chloride, potassium tellurite, nalidixic acid, aztreonam and sodium butyrate. Cell growth was inhibited by all other compounds tested within the Biolog GEN III sensitivity assays.

Results obtained with API 50 CH and API 20NE strips indicate that cells of this species oxidize or assimilate of the following substrates: L-arabinose, D-glucose, aesculin, sucrose, D-mannose, D-mannitol, potassium gluconate, trisodium citrate, capric acid and malic acid. Cells of this species exhibited the following enzymatic activities detected by API 20NE and API ZYM: reduction of nitrates to nitrites, gelatine hydrolysis, leucine arylamidase, esterase (C8) and weakly positive for esterase (C4), lipase (C14), valine arylamide, trypsin and naphtol-AS-BI-phosphohydrolase, acid phosphatase and α-fucosidase. Acids from glucose were negative. Strain B22^T^ resulted positive for the hydrolysis of Tween 20 and Tween 80 as well as for phytase and acid phosphatase. It also produced IAA and siderophores. It did not produce amylase, cellulase, caseinase, lecithinase, alkaline phosphatase and chitinase.

The main cellular fatty acids of strain B22^T^ were C_16:0_, summed feature 3 (C_16:1_ *ω*7*c*/C_16:1_ *ω*6*c*) and summed feature 8 (C_18:1_ *ω*7*c*/C_18:1_ *ω*6*c*). The major polar lipids identified were diphosphatidylglycerol and phosphatidylethanolamine, while the predominant respiratory quinone was ubiquinone (Q-9).

The genome of *P. halotolerans* B22^T^ consists of a circular chromosome of 5.7 Mbp, G+C value of 60.5 mol% and 5014 protein-coding genes. The whole genome sequence of *P. halotolerans* B22^T^ has been deposited al NCBI and is publicly available at NCBI GeneBank accs. no. JBCNTJ010000000.

The type strain is B22^T^ (= CECT31209 = LMG33902), isolated from the rhizospheric soil of the halophyte plant *Salicornia hispanica*.

## 5 Conclusions

In this study, we characterized a novel *Pseudomonas* species isolated from the rizospheric soil of the halophite plant *Salicornia hispanica*. Using a polyphasic taxonomy approach, we demonstrate that strain B22^T^ represents a novel species within the *Pseudomonas corrugata* subgroup. Furthermore, this study underscores the potential of *P. halotolerans* as a sustainable biocontrol agent, given its PGP activity in tomato plants and its ability to reduce phytopathogen virulence factors, thereby mitigating damage to fruits and plants. These attributes suggest that *P. halotolerans* could play a crucial role in enhancing plant health and reducing disease-related losses in agriculture.

## Data Availability

The datasets presented in this study can be found in online repositories. The names of the repository/repositories and accession number(s) can be found in the article/**Supplementary Material**.
