# Supplementary figures and images for: Pseudomonas halotolerans sp. nov., a halotolerant biocontrol agent with plant-growth properties
Source: Front Plant Sci. 2025 May 21;16:1605131. doi: 10.3389/fpls.2025.1605131 (PMC12133896; doi:10.3389/fpls.2025.1605131)

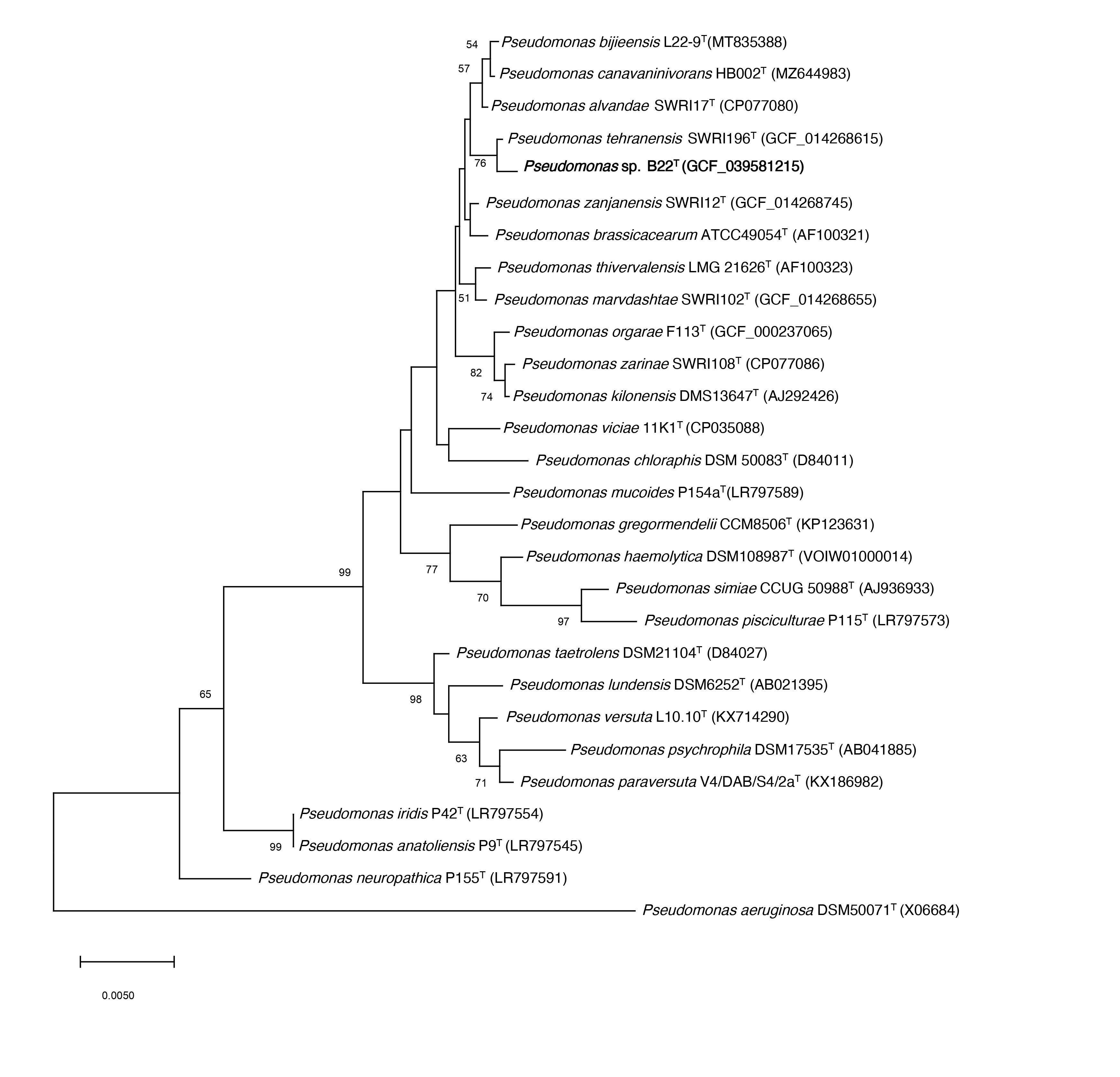

Supplement: Supplementary file 1 [file Image1.jpeg]

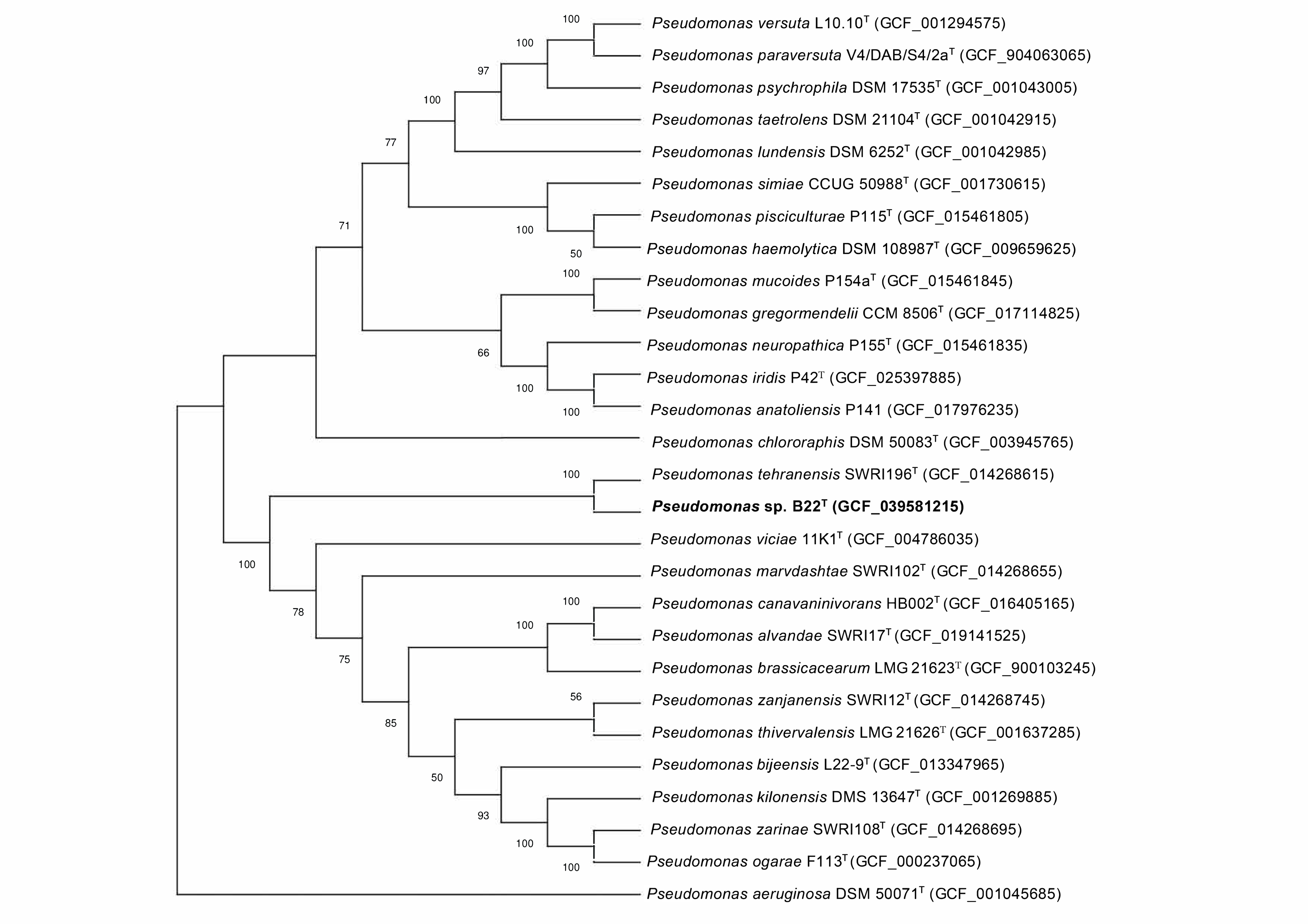

Supplement: Supplementary file 2 [file Image2.jpeg]

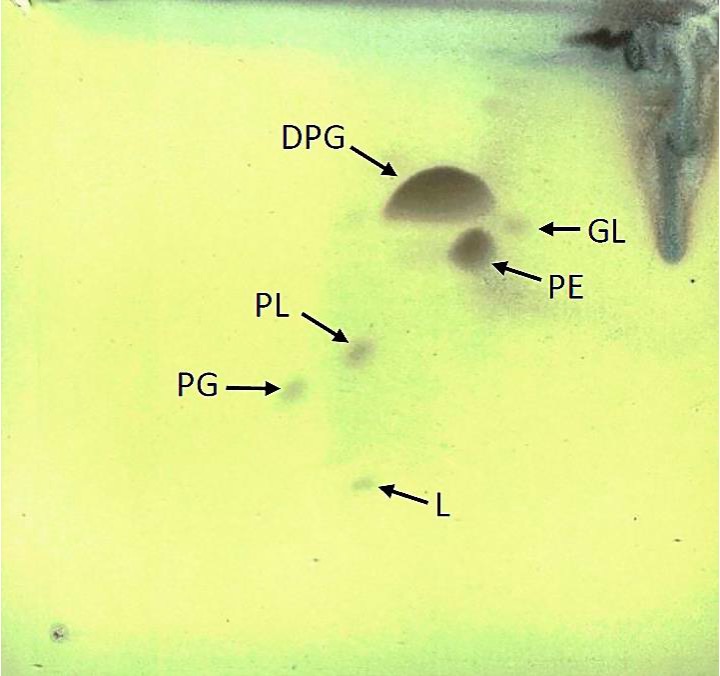

Supplement: Supplementary file 3 [file Image3.jpeg]

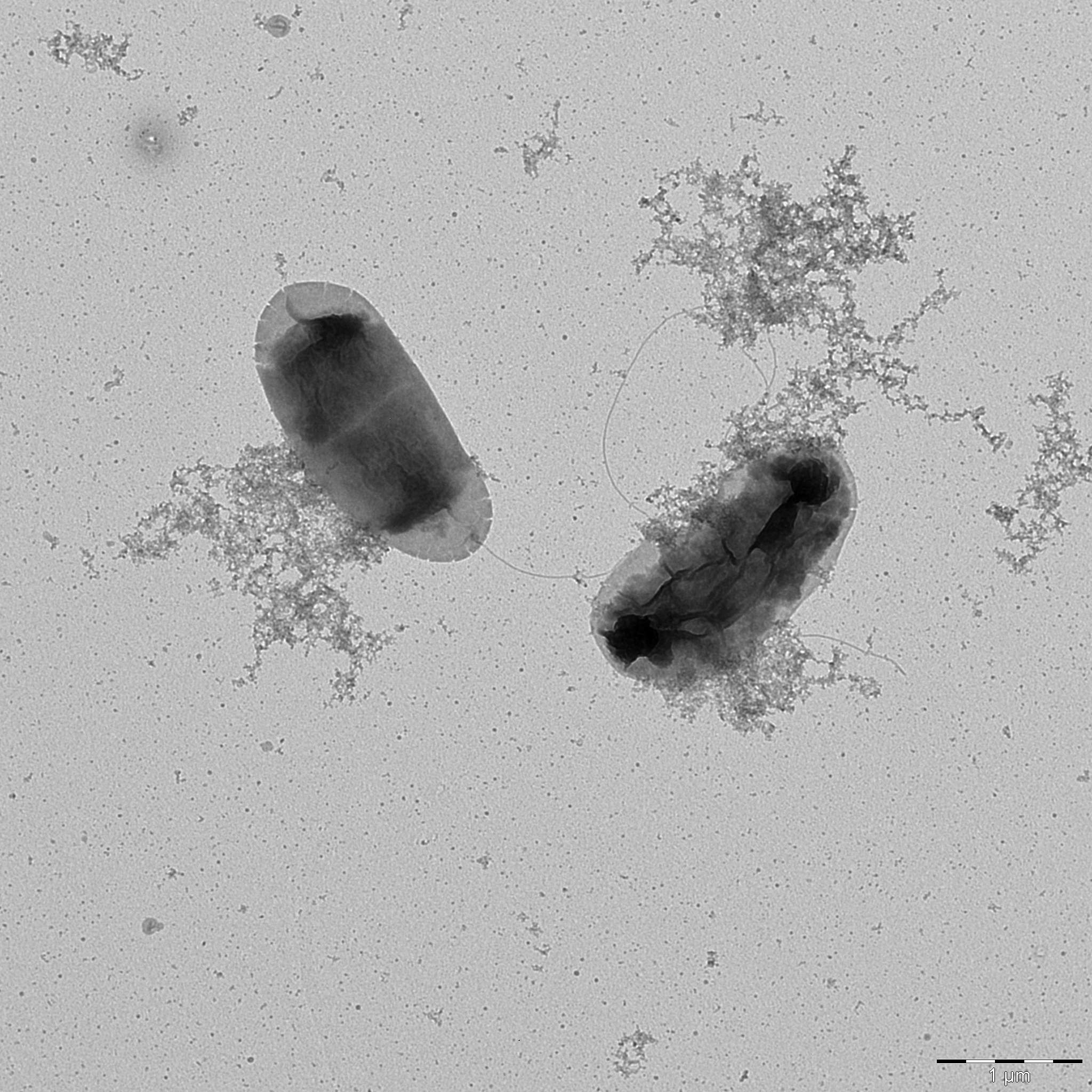

Supplement: Supplementary file 4 [file Image4.tif]

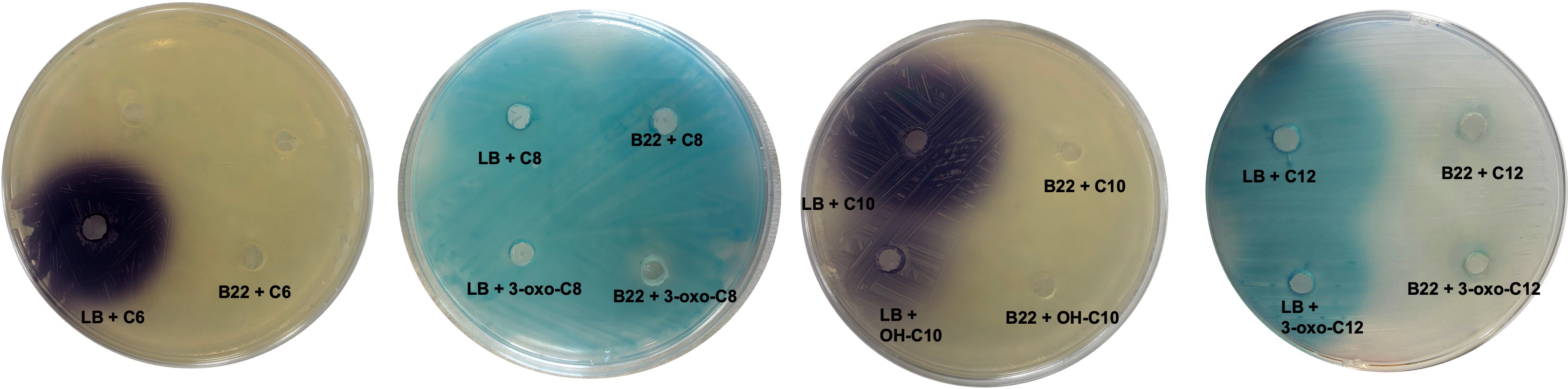

Supplement: Supplementary file 5 [file Image5.jpeg]

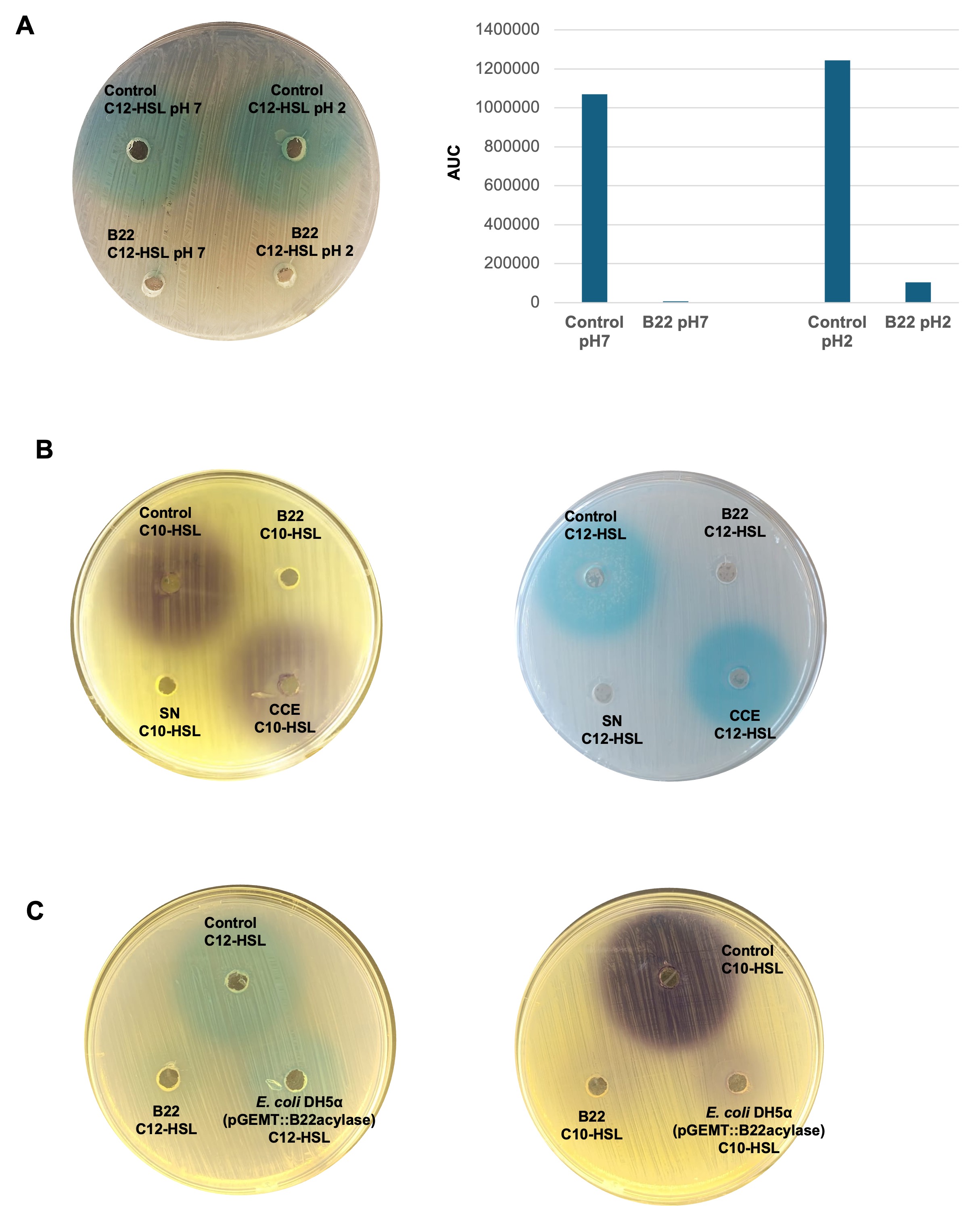

Supplement: Supplementary file 6 [file Image6.jpeg]

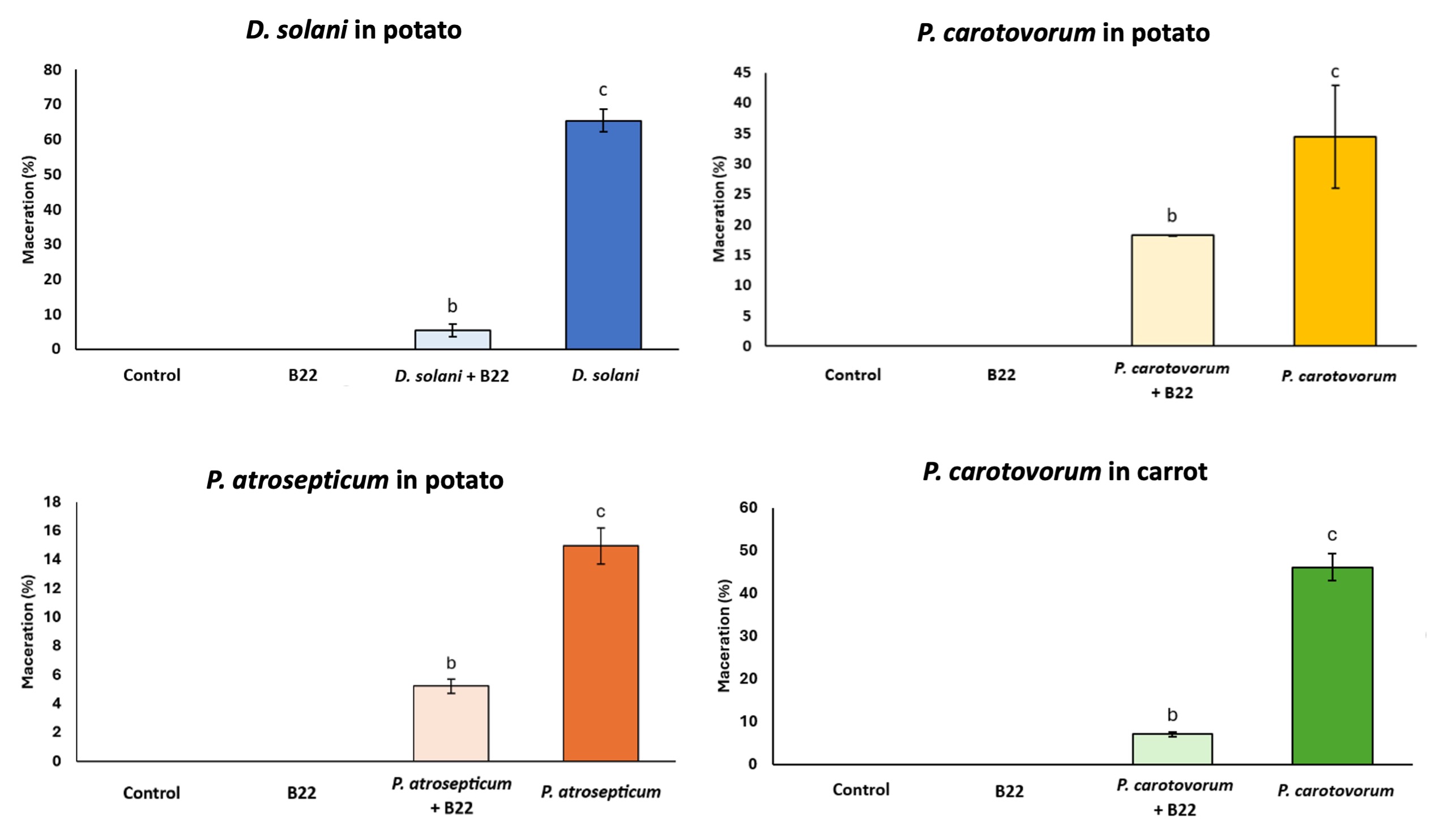

Supplement: Supplementary file 7 [file Image7.jpeg]

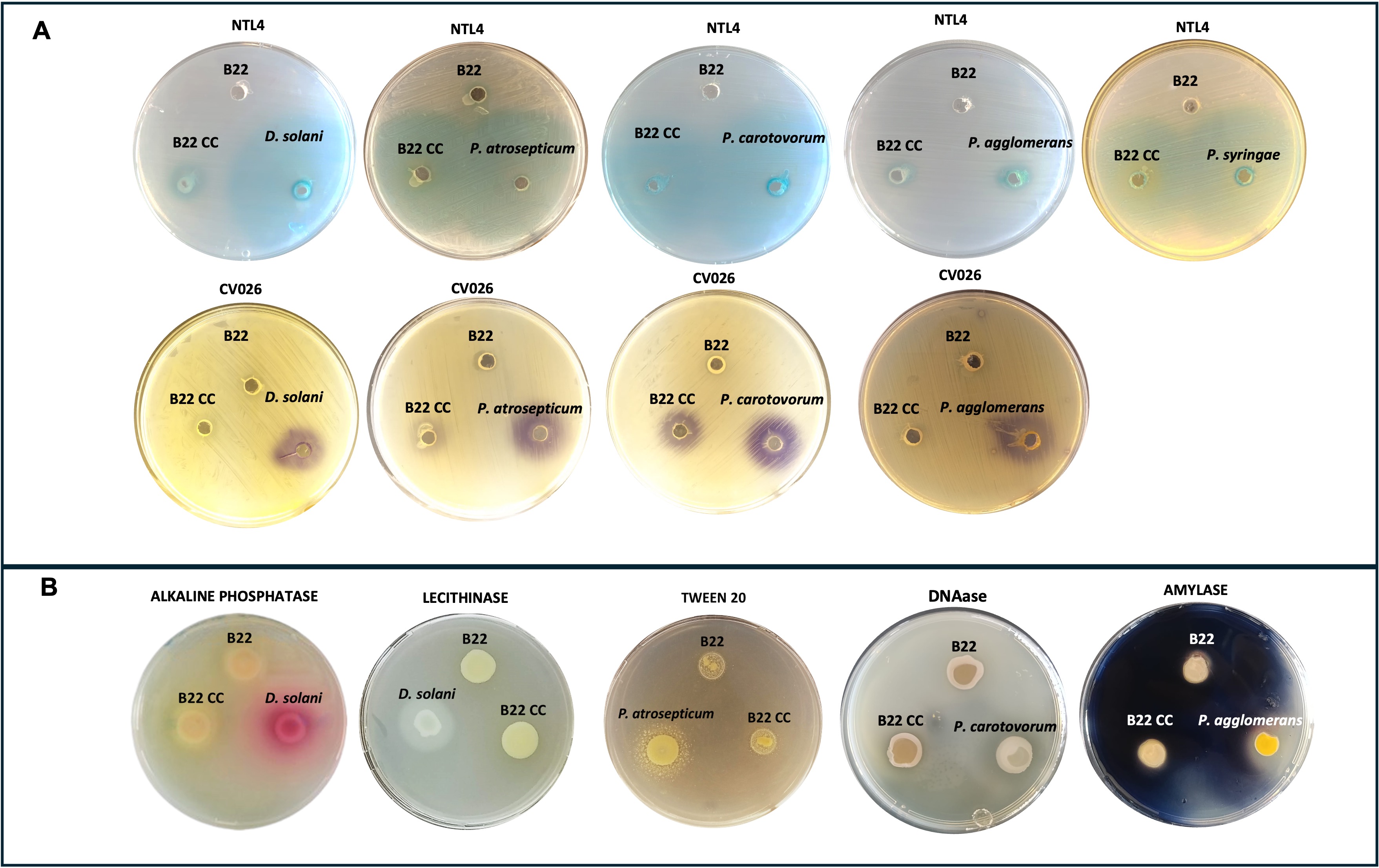

Supplement: Supplementary file 8 [file Image8.jpeg]
