# Supplementary material for: Pseudomonas halotolerans sp. nov., a halotolerant biocontrol agent with plant-growth properties
Source: Front Plant Sci. 2025 May 21;16:1605131. doi: 10.3389/fpls.2025.1605131 (PMC12133896; doi:10.3389/fpls.2025.1605131)
Supplement: Supplementary file 10 [file Table2.docx]

Supplementary Material

*Pseudomonas* *halotolerans* sp. nov., A Halotolerant Biocontrol Agent with Plant-Growth Properties

Patricia Sánchez^1^, Inés Castillo^1^, Fernando Martínez-Checa^1,2^, Inmaculada Sampedro^1,2*^, Inmaculada Llamas^1,2^

^1^Department of Microbiology, Faculty of Pharmacy, University of Granada, Granada, Spain
^2^Biomedical Research Centre (CIBM), Institute of Biotechnology, University of Granada, Granada, Spain

*** Correspondence:** [isampedro@ugr.es](mailto:isampedro@ugr.es)

**SUPPLEMENTARY TABLES**

**Supplementary Table S1.** Genome sequence similarity between strain B22^T^ and closely related type strains with available genomes.

**Supplementary Table S2.** Phenotypic characterization of the strain B22^T^ and their closest species.

**Supplementary Table S3.** Phenotypic characteristics of strain B22^T^ and plant pathogens.

**SUPPLEMENTARY FIGURES**

**Supplementary Figure S1.** Neighbour-joining phylogenetic tree based on 16S rRNA gene sequences showing the position of strains B22^T^ (bold) and its relationship with other *Pseudomonas* related species. The GenBank/EMBL/DDBJ accession number of each sequence is shown in parenthesis. Bootstrap values are expressed as percentages of 1,000 replications, and those >50% are shown at branch points. *P. aeruginosa* DSM50071^T^ was used as an outgroup. Bar, 0.005 substitutions per nucleotide position.

**Supplementary Figure S2.** Phylogenetic tree reconstructed by the neighbor-joining method based on four concatenated gene sequences (16S rRNA, gyrB, rpoD and rpoB) of strain B22^T^ and 26 Pseudomonas related type strains. The robustness of tree topologies was evaluated with 1000 bootstrap replications, and values of >50% are shown at the nodes of the branches. The strain Pseudomonas aeruginosa DSM50071*^T^ served as outgroup.*

**Supplementary Figure S3.** Polar lipid profile of strain B22^T^ determined after two-dimensional TLC using molybdatophophoric acid. DPG: diphosphatidylglycerol, PE: phosphatidylethanolamine, PG: phosphatidylglycerol, GL: glycolipid, PL: phospholipid, L: lipid.

**Supplementary Figure S4.** Transmission micrograph of cells of strain B22^T^.

**Supplementary Figure S5.** Detection of AHL degradation activity using a diffusion agar-plate method and the biosensors *C.* *subtsugae* CV026, *C. violaceum* VIR07 and *A.* *tumefaciens* NTL4 (pZLR4). Supernatant of 24h incubation culture of strain B22^T^ in LB medium supplemented with 10 µM of each AHL. Controls contains LB medium supplemented with the same concentration of each AHL.

**Supplementary Figure S6. Characterization of strain B22^T^ quorum quenching activity. a.** Remaining C12-HSL after 24h of incubation with strain B22^T^ under neutral and acidic conditions by diffusion agar-plate assay using the biosensor NTL4 (left) and HPLC/MS (right). HPLC values as referred to as area under the curve (AUC). **b.** Remaining C10-HSL and C12-HSL in the culture, supernatant (SN) and crude cellular extract (CCE) of strain B22^T^ by difusión agar-plate assay using the biosensor VIR07 and NTL4**. c.** Remaining C10-HSL and C12-HSL of strain B22^T^ acylase cloned into pGEM-T after heterologous expression in *E. coli* DH5α using the biosensors NTL4 and VIR07. Controls contain cell-free LB medium supplemented with 10 µM of each AHL.

**Supplementary Figure S7.** Detection AHLs (A) and phenotypes (B) in the culture and co-cultures of strain B22^T^ and the pathogens. The biosensors *C.* *subtsugae* and *A.* *tumefaciens* NTL4 (pZLR4) were used to detect AHLs.

**Supplementary Figure S8.** Interference on maceration originated by pathogens in potato and carrots by the cocultivation of B22^T^. Different letters indicate statistically significative differences (p < 0.01).
